# Bioengineered Model of Human LGMD2B Skeletal Muscle Reveals Roles of Intracellular Calcium Overload in Contractile and Metabolic Dysfunction in Dysferlinopathy

**DOI:** 10.1002/advs.202400188

**Published:** 2024-06-17

**Authors:** Alastair Khodabukus, Neel K. Prabhu, Taylor Roberts, Meghan Buldo, Amber Detwiler, Zachary D. Fralish, Megan E. Kondash, George A. Truskey, Timothy R. Koves, Nenad Bursac

**Affiliations:** ^1^ Department of Biomedical Engineering Duke University Durham NC 27708 USA; ^2^ Duke Molecular Physiology Institute Duke University Durham NC 27708 USA

**Keywords:** calcium, dysferlin, limb girdle muscular dystrophy 2B/2R, lipid droplet, mitochondria, skeletal muscle, tissue engineering

## Abstract

Dysferlin is a multi‐functional protein that regulates membrane resealing, calcium homeostasis, and lipid metabolism in skeletal muscle. Genetic loss of dysferlin results in limb girdle muscular dystrophy 2B/2R (LGMD2B/2R) and other dysferlinopathies – rare untreatable muscle diseases that lead to permanent loss of ambulation in humans. The mild disease severity in dysferlin‐deficient mice and diverse genotype‐phenotype relationships in LGMD2B patients have prompted the development of new in vitro models for personalized studies of dysferlinopathy. Here the first 3‐D tissue‐engineered hiPSC‐derived skeletal muscle (“myobundle”) model of LGMD2B is described that exhibits compromised contractile function, calcium‐handling, and membrane repair, and transcriptomic changes indicative of impaired oxidative metabolism and mitochondrial dysfunction. In response to the fatty acid (FA) challenge, LGMD2B myobundles display mitochondrial deficits and intracellular lipid droplet (LD) accumulation. Treatment with the ryanodine receptor (RyR) inhibitor dantrolene or the dissociative glucocorticoid vamorolone restores LGMD2B contractility, improves membrane repair, and reduces LD accumulation. Lastly, it is demonstrated that chemically induced chronic RyR leak in healthy myobundles phenocopies LGMD2B contractile and metabolic deficit, but not the loss of membrane repair capacity. Together, these results implicate intramyocellular Ca^2+^ leak as a critical driver of dysferlinopathic phenotype and validate the myobundle system as a platform to study LGMD2B pathogenesis.

## Graphical abstract

In this study, a hiPSC‐derived 3D tissue‐engineered model of LGMD2B skeletal muscle is developed and applied to show that calcium handling abnormalities drive multiple contractile and metabolic deficits in dysferlinopathy. This human in vitro model replicates transcriptional signatures and drug responses of diseased muscles from patients and small animal models, highlighting its utility for future mechanistic and drug development studies.

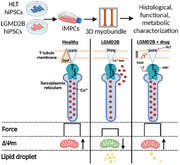

## 1 Introduction

Dysferlin deficiency leads to the development of several rare progressive muscular dystrophies such as limb girdle muscular dystrophy 2B/2R (LGMD2B/2R) and Miyoshi myopathy (MM), collectively known as dysferlinopathies.^[^ ^1^, ^2^ ^]^ In humans, dysferlinopathies have late‐teen onset in select proximal or distal limb‐girdle muscles, followed by progressive muscle weakness^[^ ^3^, ^4^ ^]^ and significant muscle replacement by fat,^[^ ^5^, ^6^, ^7^ ^]^ eventually resulting in permanent loss of ambulation. Enriched in skeletal muscle, dysferlin is a multi‐functional protein that regulates membrane resealing,^[^ ^8^, ^9^ ^]^ vesicle trafficking,^[^ ^9^ ^]^ and transverse‐tubule (T‐tubule) formation and function.^[^ ^10^, ^11^, ^12^, ^13^ ^]^ Specifically, dysferlin enables optimal coupling of the voltage‐gated dihydropyridine receptor (DHPR) Ca^2+^ channel in sarcolemma and the ryanodine receptor 1 (RyR1) Ca^2+^ release channel in sarcoplasmic reticulum (SR).^[^ ^10^, ^12^ ^]^ Loss of dysferlin results in cytosolic Ca^2+^ overload, diminished Ca^2+^ transient in uninjured muscle, and increases Ca^2+^ flux during membrane damage that yields deficient membrane repair.^[^ ^8^, ^9^ ^]^ Dysferlin is also essential for skeletal muscle lipid homeostasis, although how dysferlin regulates lipid metabolism is currently unknown. During disease progression, lipid droplets (LDs) accumulate within myofibers^[^ ^5^, ^11^ ^]^ and ultimately myofibers are replaced by adipocytes.^[^ ^14^ ^]^ The contribution of altered Ca^2+^ handling to lipid abnormalities in dysferlinopathy is currently unclear.

Currently, there are no approved therapies that can cure dysferlinopathy, or slow or revert disease progression. The development of effective therapies for dysferlinopathy is hindered by the mild disease symptoms in dysferlin‐deficient mouse models. Dysferlin‐deficient mice recapitulate clinical histopathological features such as myofiber necrosis,^[^ ^11^, ^15^ ^]^ immune cell infiltration,^[^ ^15^, ^16^ ^]^ and steatosis^[^ ^5^, ^17^ ^]^ in a subset of muscles. However, they typically do not display severe muscle weakness and loss of ambulation seen in humans.^[^ ^15^, ^18^, ^19^, ^20^ ^]^ Accurate modeling of human dysferlinopathies in animal models is further complicated by the lack of mutational hotspots, and the poor correlation of patient genotypes with disease progression and severity.^[^ ^21^, ^22^ ^]^ Combined with generally low translational success of drug candidates identified in small animal models,^[^ ^23^ ^]^ there is a clear need to develop in vitro human LGMD2B models for patient‐specific disease modeling and drug discovery.

Traditional in vitro LGMD2B models have been performed in 2D monolayers from primary^[^ ^24^, ^25^ ^]^ or immortalized^[^ ^26^, ^27^ ^]^ human myoblasts and primary mouse myoblasts.^[^ ^28^, ^29^ ^]^ While impaired myoblast differentiation and increased susceptibility to injury have been observed in LGMD2B monolayers, these cultures allow for only short experiments (7‐10 days) due to cellular detachment and do not support measurements of muscle function (i.e. force generation and fatigue).^[^ ^30^, ^31^ ^]^ Additionally, the difficulties with obtaining patients’ biopsies, the limited expansion potential of primary muscle cells,^[^ ^32^ ^]^ and genetic and functional abnormalities and variability of immortalized cell lines limit the translational relevance of studies using these cells.^[^ ^33^ ^]^ Conversely, human induced pluripotent stem cells (hiPSCs) represent an unlimited and versatile cell source for in vitro disease modeling studies.^[^ ^31^, ^34^ ^]^ Previously, we have utilized hiPSC‐derived^[^ ^35^ ^]^ and primary^[^ ^36^ ^]^ muscle progenitor cells to generate the first functional 3D human skeletal muscle tissues (“myobundles”) that display physiological responses to drugs,^[^ ^36^, ^37^, ^38^ ^]^ toxins,^[^ ^39^, ^40^ ^]^ and exercise^[^ ^41^, ^42^ ^]^ and can be used to model genetic^[^ ^37^ ^]^ and acquired^[^ ^41^ ^]^ human muscle disease.

In the present study, we demonstrate the first 3D hiPSC‐derived myobundle model of LGMD2B, which similar to clinical symptoms, displays abnormal muscle function, Ca^2+^ handling, metabolism, and membrane repair. LGMD2B myobundles exhibit downregulation of gene sets involved in intracellular Ca^2+^ regulation, oxidative metabolism, and mitochondrial function. In response to fatty acid (FA) treatment, LGMD2B myobundles show decreased mitochondrial membrane potential and failure to increase oxygen consumption. Treatment with the RyR inhibitor dantrolene (DNT) or the novel glucocorticoid vamorolone (VAM) restores LGMD2B contractile function, improves membrane repair, and reduces LD accumulation. Chemically inducing an RyR Ca^2+^ leak in healthy (HLT) myobundles recapitulates contractile and metabolic abnormalities found in LGMD2B tissues. Together, these results implicate cytosolic Ca^2+^ overload as a critical driver in LGMD2B intramuscular pathology and validate the myobundle system as a novel 3D platform to study pathogenesis of LGMD2B.

## 2 Results

### 2.1 Generation and Differentiation of LGMD2B hiPSC Myobundles

**Figure 1:**
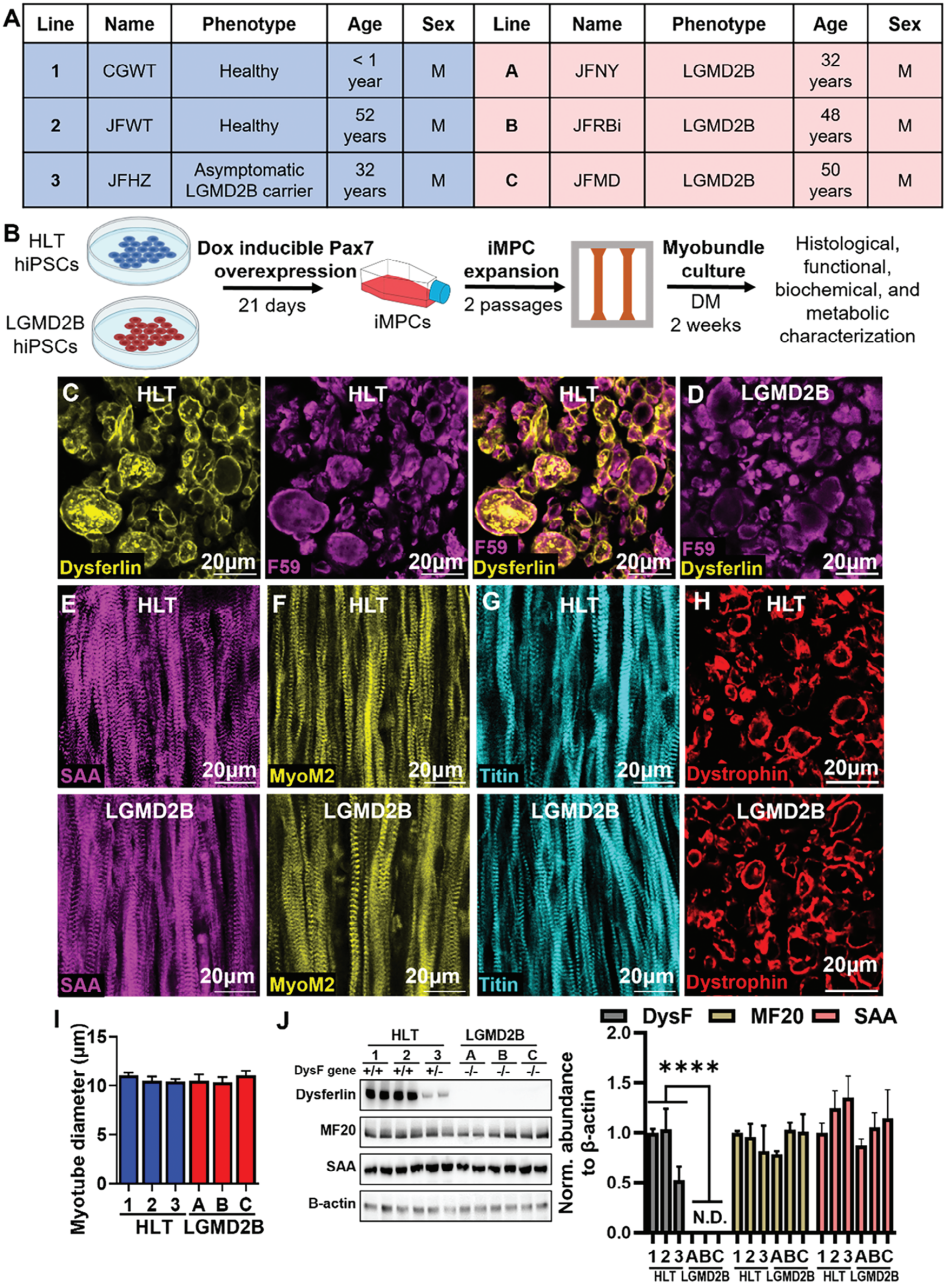
Myogenesis in healthy and LGMD2B myobundles. A) Information and genotype for healthy (HLT, 1, 2, 3) and LGMD2B (A, B, C) hiPSC lines used in all presented studies. B) Schematic of experimental flow from iMPC generation to myobundle fabrication and assessment. C,D) Representative cross‐sections of C) healthy and D) LGMD2B myobundles stained for dysferlin and fast myosin heavy chain (F59). E–G) Representative whole‐myobundle staining of E) sarcomeric alpha‐actinin (SAA), F) myomesin‐2 (MyoM2), and G) titin. H,I) Representative cross‐sections for H) dystrophin staining used for quantification of myotube diameter I, n = 6 myobundles per donor). J) Representative western blots and quantified protein expression (normalized to β‐actin) of dysferlin and myosin heavy chain (MF20), (N = 4 differentiations per donor, 6 pooled myobundles per differentiation). ^****^ *P* < 0.0001 vs. HLT. Data: mean ± SEM.

Dysferlinopathy is inherited in an autosomal recessive pattern, such that heterozygous individuals are healthy carriers. To generate a 3D in vitro model of LGMD2B, we first applied a doxycycline‐inducible Pax7 overexpression system to generate an expandable population of induced myogenic progenitor cells (iMPCs) from 3 healthy (including one healthy heterozygous sibling, line 3) and 3 LGMD2B hiPSC lines (**Figure** **1**A). The loss of dysferlin did not impact iMPC generation, as evident from similar proportions of healthy and LGMD2B iMPCs expressing muscle stem cell transcription factors Pax7 or Myf5 (Figure S1, Supporting Information). Since defective myogenic differentiation has been reported in primary LGMD2B human MPCs,^[^ ^25^ ^]^ we assessed HLT and LGMD2B iMPC differentiation capacity in 2D monolayers and found no significant differences in sarcomeric alpha‐actinin (SAA) area or proportion of nuclei expressing the terminal differentiation transcription factor myogenin (MyoG) (Figure S2, Supporting Information). We then utilized HLT and LGMD2B iMPCs to generate 3D myobundles by embedding iMPCs in a fibrin/Matrigel composite hydrogel anchored to a velcro frame (Figure 1B; Figure S3A,B, Supporting Information), as we have previously described.^[^ ^35^ ^]^ Both HLT and LGMD2B myobundles underwent typical cell‐mediated compaction during the first 4 days of culture, followed by a gradual increase in tissue size due to both iMPC proliferation and myotube hypertrophy (Figure S3C, Supporting Information). Live/dead staining revealed no difference in cell viability over time between HLT and LGMD2B myobundles (Figure S3D, Supporting Information), providing a platform to dissect the consequences of dysferlin deficiency. As characteristic for native muscle,^[^ ^9^ ^]^ dysferlin in 2‐wk HLT myobundles predominantly resided in the plasma membrane (Figure 1C), as well as in intracellular puncta resembling native dysferlin‐positive vesicles.^[^ ^43^ ^]^ On the other hand, LGMD2B myobundle cross‐sections did not show dysferlin expression (Figure 1D). Similar to findings in 2D cultures (Figure S2, Supporting Information), no significant differences in nuclei number, myobundle cross‐sectional area, or F‐actin^+^ myotube area were found between healthy and LGMD2B myobundles (Figure S4, Supporting Information). In human muscle, dysferlin binds directly to the M‐band protein myomesin‐2 (MyoM2)^[^ ^44^ ^]^ and forms a protein complex with titin,^[^ ^45^ ^]^ suggesting that sarcomere structure could be impaired in LGMD2B myobundles. However, we observed mature cross‐striation patterning of SAA, MyoM2, and titin in both HLT and LGMD2B myobundles (Figure 1E–G) and found no difference in myotube diameter based on dystrophin staining (Figure 1H,I). Further confirming LGMD2B phenotype, dysferlin protein was not detected in LGMD2B myobundles and was only at 50% of HLT level in myobundles made from the heterozygous carrier line (HLT, line 3, Figure 1J). In agreement with our immunohistological analysis, similar protein abundance of the sarcomeric proteins myosin heavy chain and SAA were found by western blot in HLT and LGMD2B myobundles. Overall, these studies indicated no differences in muscle differentiation or morphology between healthy and LGMD2B myobundles.

### 2.2 Functional Characterization of LGMD2B Myobundles

**Figure 2:**
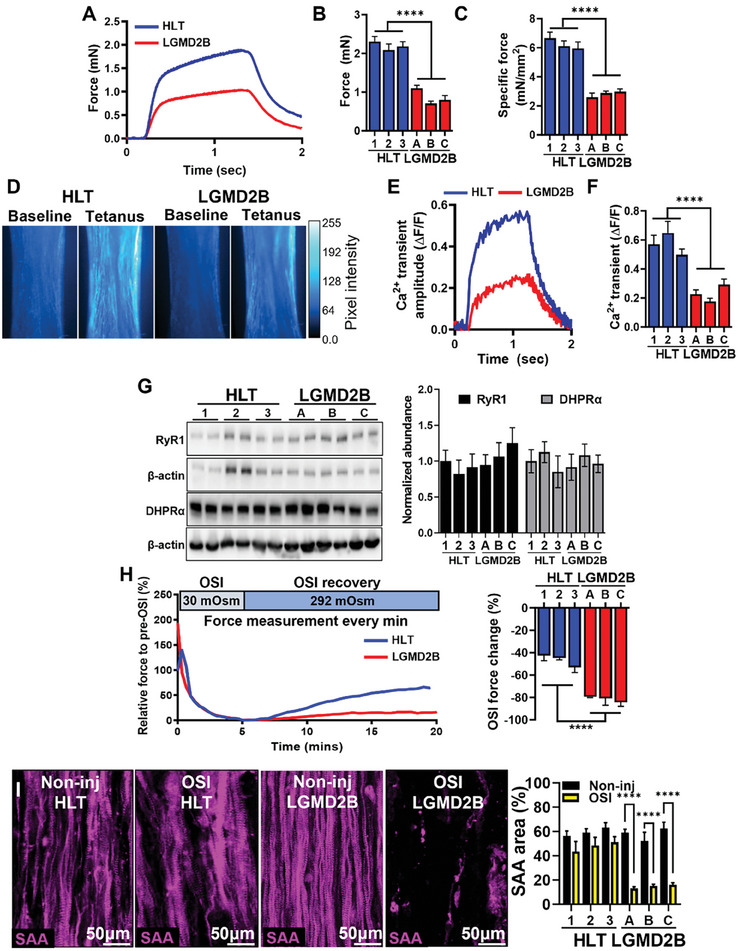
Functional characterization of healthy and LGMD2B myobundles. A) Representative tetanic force traces and quantifications of B) force (n = 16‐18 per donor) and C) specific force (n = 6 per donor) generation in healthy (HLT) and LGMD2B myobundles. D–F) Representative D) Fluo‐8 AM fluorescence intensity images at baseline and peak tetanic contraction, E) corresponding Ca^2+^ transient traces, and F) quantified Ca^2+^ transient amplitudes (ΔF/F of Fluo‐8, n = 6–8 per donor). G) Representative western blots and quantified protein abundance (normalized to β‐actin) of ryanodine receptor 1 (RyR1) and dihydropyridine receptor α (DHPRα), (N = 4 differentiations per donor, 6 pooled myobundles per differentiation) H) Representative traces and quantification of force change before and after osmotic shock injury (OSI, n = 6 myobundles per donor). I) Representative immunostaining of SAA pre‐ and post‐OSI and corresponding quantification of percent SAA^+^ area. ^****^ *P* < 0.0001 in LGMDB vs. HLT or non‐injured vs. OSI (in I).

To determine if despite similar structures LGMD2B and HLT myobundles are functionally different, we performed isometric contractile force measurements and found a ≈50% decrease in peak tetanic and specific force amplitude in LGMD2B tissues (**Figure** **2**A–C). Furthermore, in agreement with mouse studies,^[^ ^10^ ^]^ Ca^2+^ transient amplitude was reduced in LGMD2B vs. HLT myobundles (Figure 2D–F; Movie S1, Supporting Information) at a similar degree as the measured contractile deficit. The reduced Ca^2+^ transient amplitude was not caused by a lower abundance of ryanodine receptor 1 (RyR1) or the dihydropyridine receptor alpha 1 (DHPRα, Figure 2G), critical regulators of electrically stimulated Ca^2+^ release in skeletal muscle.^[^ ^46^ ^]^ Rather, these results were consistent with dysferlin deficiency‐induced SR Ca^2+^ leak leading to decreases in SR Ca^2+^ stores and Ca^2+^ transient amplitude.^[^ ^10^, ^12^ ^]^ To additionally test if LGMD2B myobundles exhibited impaired Ca^2+^‐sensitive membrane resealing and repair, we developed an in vitro model of hypo‐osmotic shock injury (OSI, Figure 2H). Specifically, HLT and LGMD2B myobundles were incubated in a ≈30mOsm hypo‐osmotic media for 5 min and allowed to recover in normo‐osmotic media for an additional 15 min, with force generation assessed every minute. This test revealed a significant decrease in post‐OSI force recovery in LGMD2B compared to healthy myobundles (Figure 2H), which was accompanied by an almost complete loss of myotubes (Figure 2I). Overall, these results confirmed that the LGMD2B myobundle model successfully replicated deficits in calcium handling and membrane repair observed in disease‐affected dysferlin‐deficient muscles in vivo.

### 2.3 RNA‐Sequencing Analysis of LGMD2B Myobundles

**Figure 3:**
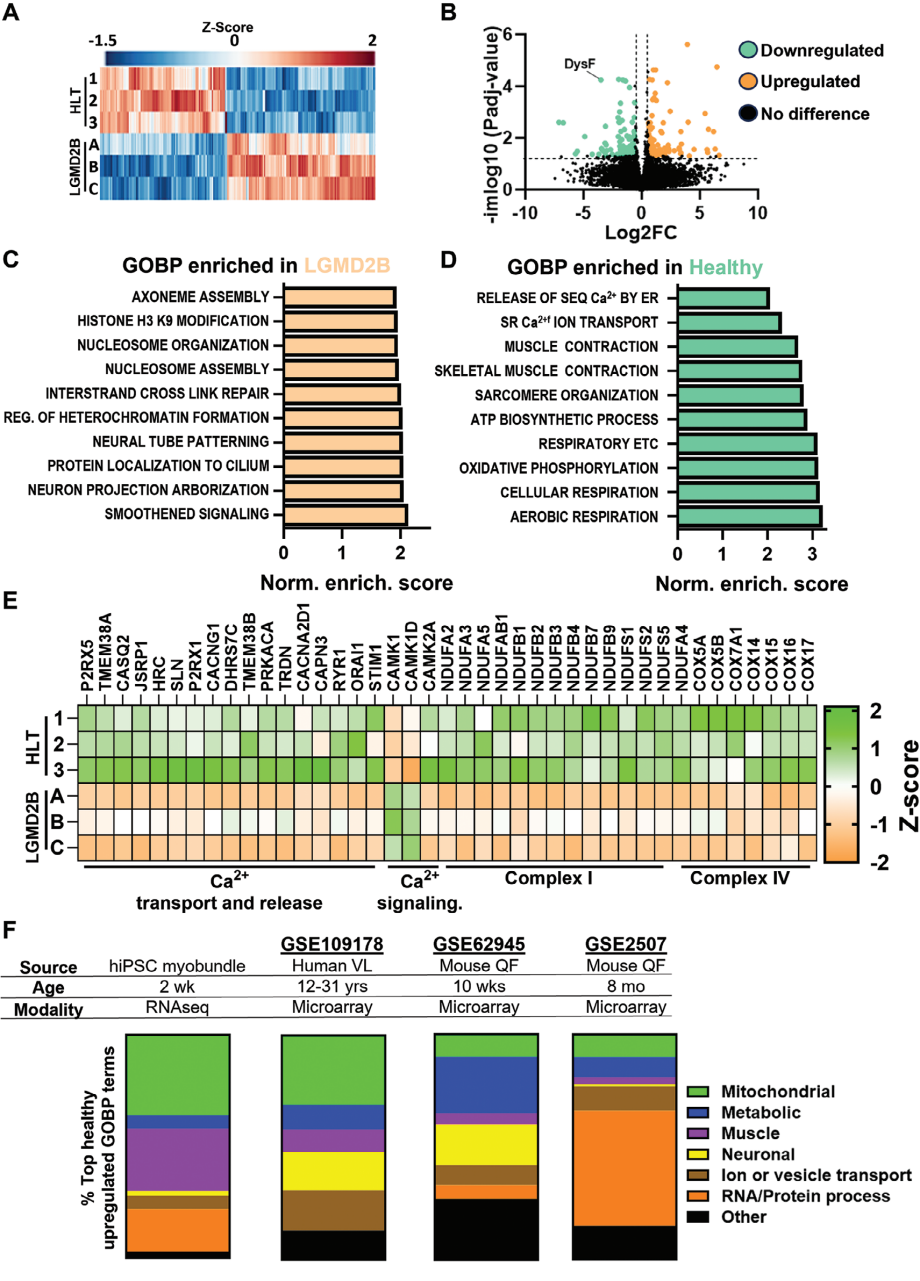
Transcriptomic comparisons of healthy and LGMD2B myobundles and native muscle. A,B) RNA‐seq (A) heatmap and (B) volcano plot of genes with significantly different (Padj < 0.05, |log2FC| ≥ 1) expression in LGMD2B vs. healthy myobundles. C,D) GOBP terms (identified by GSEA on RNA‐seq data) enriched in (C) healthy and (D) LGMD2B myobundles against normalized enrichment score (FDR < 0.15). E) Expression heatmaps of selected genes related to calcium handling and mitochondrial biological processes in LGMD2B vs. HLT myobundles. F) Comparison of GOBP terms upregulated in RNA‐seq analysis from healthy vs. LGMD2B myobundles and microarray datasets from native symptomatic human and presymptomatic (10 wks) and symptomatic (8 mo) mouse healthy vs. LGMD2B muscles.

To gain further insights into transcriptomic mechanisms of the disease, we performed RNA‐sequencing (RNA‐seq) analysis of 2‐wk healthy and LGMD2B myobundles (**Figure** **3**A) and found 147 differentially expressed genes (|log2(fold‐change)| ≥ 1, Padj < 0.05, Figure 3B). Gene set enrichment analysis (GSEA), using the gene ontology biological process (GOBP) annotation revealed expected and novel significantly (P < 0.05) altered biological processes including upregulation of smoothened signaling (GO:0007224), protein localization to cilium (GO:0061512), nucleosome organization (GO:0034728), DNA repair (GO:0036297), and neural tube patterning (GO:0021532) in LGMD2B myobundles (Figure 3C). On the other hand, similar to previous human^[^ ^47^ ^]^ and mouse^[^ ^48^, ^49^ ^]^ studies, healthy myobundles had significantly upregulated muscle contraction (GO:0006936) and Ca^2+^‐handling processes, including sarcoplasmic reticulum Ca^2+^ ion transport (GO:0070296) and regulation of RyR‐sensitive Ca^2+^ release channel activity (GO:0060314) (Figure 3D). However, the strongest differential gene signature was related to aerobic respiration (GO:0009060) and oxidative phosphorylation (GO:0006119). These findings were further confirmed by expression analysis of individual calcium transport, release, signaling, and mitochondrial complex I and IV genes (Figure 3E). We then compared our RNA‐seq results to previously published human^[^ ^47^ ^]^ and mouse^[^ ^48^, ^49^ ^]^ DNA microarray datasets. Since RNA‐seq (the whole transcriptome) and DNA microarray (selected transcripts) datasets cannot be directly compared or integrated, we first performed GSEA in each dataset to identify differentially regulated GOBP terms. As different GOBPs are generated by specific gene sets, we then manually classified each GOBP term into a general classification scheme based on cell function, which in turn allowed us to compare differentially expressed GOBPs between datasets (Figure 3F). Consistent with LGMD2B myobundle results, human patient samples (GSE109178) exhibited a strong metabolic and mitochondrial gene expression signature (Figure S5, Supporting Information). Moreover, presymptomatic (10 wk old, GSE62945)^[^ ^49^ ^]^ and symptomatic (8‐month‐old, GSE2507)^[^ ^48^ ^]^ dysferlin‐deficient mouse samples showed significantly downregulated genes related to mitochondrial and other metabolic processes (Figure S6, Supporting Information). Unlike myobundles, human and mouse LGMD2B muscles showed highly upregulated immunological processes such as antigen processing and presentation (GO:0019882) and positive regulation of type I interferon production (GO:0032481) (Figures S5C,S6B,D,S7, Supporting Information). This difference likely reflected the strong immune cell infiltration seen in native dysferlin‐deficient muscle,^[^ ^15^, ^16^ ^]^ not modeled in LGMD2B myobundles. However, similar to myobundle results, biological processes related to basement membrane organization, cilia assembly, and neuronal organization were all significantly upregulated in native LGMD2B muscles (Figure S7C, Supporting Information).

### 2.4 Effect of Fatty Acids on LGMD2B Metabolism and Lipid Droplet Formation

**Figure 4:**
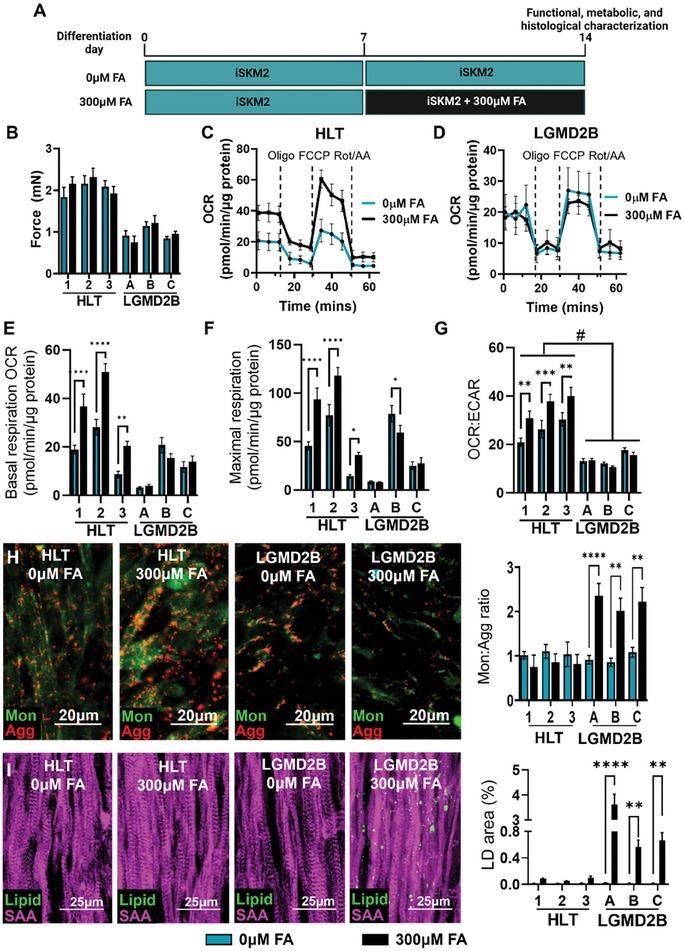
Mitochondrial dysfunction and lipid accumulation in LGMD2B myobundles. A) Schematic of experimental flow to assess effects of fatty acid (FA) supplementation on myobundles. B) Tetanic force generation following 0 and 300 µm FA (n = 4 myobundles per group). C–G) Representative Seahorse XFe24 analyzer oxygen consumption rate (OCR) traces in (C) healthy (HLT) and (D) LGMD2B myobundles and quantification of E) basal respiration, F) maximal respiration, and G) OCR:ECAR (n = 8–10 myobundles per group). H) Representative images of JC‐10 staining in healthy and LGMD2B myobundles cultured in 0 or 300 µm FA and corresponding quantification of the ratio of green monomer (mon) to red aggregate (agg) area (n = 12 per group). I) Representative immunostaining of LipidSpot (Lipid) and SAA and quantification of lipid droplet (LD) area (n = 12 per group). ^*^ *P* < 0.05, ^**^ *P* < 0.01, ^***^ *P* < 0.001, ^****^ *P* < 0.0001 vs. 0 µm FA. Data: mean ± SEM.

The strong transcriptomic signatures of mitochondrial and oxidative metabolism in RNA‐seq analysis prompted us to assess the metabolic function of LGMD2B myobundles. Specifically, serum‐free media lacking fatty acids (FAs) used for muscle differentiation was supplemented with 300 µm FAs (100 µm oleic, 100 µm linoleic, and 100 µm palmitic acid) for the last 7 days of culture to provide a natural lipid substrate for mitochondrial respiration (**Figure** **4**A). The FA addition did not impact contractile force generation of HLT or LGMD2B myobundles (Figure 4B), but differentially affected their cellular bioenergetics (Figure 4C,D). Without FAs, oxygen consumption rate (OCR), a marker of mitochondrial respiration, was similar in HLT and LGMD2B myobundles; however, with FA treatment, healthy but not LGMD2B myotubes increased basal and maximal respiration (Figure 4C–F). Measurements of anaerobic glycolysis via extracellular acidification rate (ECAR) revealed greater relative levels of oxidative phosphorylation (OXPHOS) to glycolysis as assessed by increased OCR:ECAR ratio in healthy vs. LGMD2B myobundles, which was further enhanced with FA treatment (Figure 4G). Similarly, impaired FA responsiveness (Figure S8A–C, Supporting Information) and increased glycolytic metabolism (Figure S8D–F, Supporting Information) were also detected in 8‐day LGMD2B myotubes treated with FAs for the last 4 days of 2D culture. Together, this data indicates the inability of dysferlin‐deficient muscle to adapt to an FA challenge by increased OXPHOS and an increased reliance on glycolysis to meet energy demands. To further determine if mitochondrial dysfunction may contribute to the observed metabolic deficiency in LGMD2B myobundles, we measured mitochondrial membrane potential (MMP) with JC‐10 dye (Figure 4H) and found that LGMD2B but not HLT myobundle mitochondria lost their MMP as evident from the loss of red JC‐10 aggregates which only accumulate in healthy respiring mitochondria. Since lipid droplets (LDs) accumulate within dysferlin‐deficient myofibers in vivo,^[^ ^5^ ^]^ we also utilized the fluorogenic dye LipidSpot^TM^ to detect neutral lipids and assess LD accumulation (Figure 4I). In the absence of FAs, LDs were rarely detected in myobundles; however, the addition of FAs resulted in significant accumulation in LDs specifically in LGMD2B but not HLT myobundles (Figure 4I). Together, these results indicate that dysferlin‐deficiency in LGMD2B myobundles leads to FA‐induced mitochondrial dysfunction and LD accumulation.

### 2.5 The Roles of Ca^2+^ Overload in LGMD2B Revealed by Drug Testing in Myobundles

**Figure 5:**
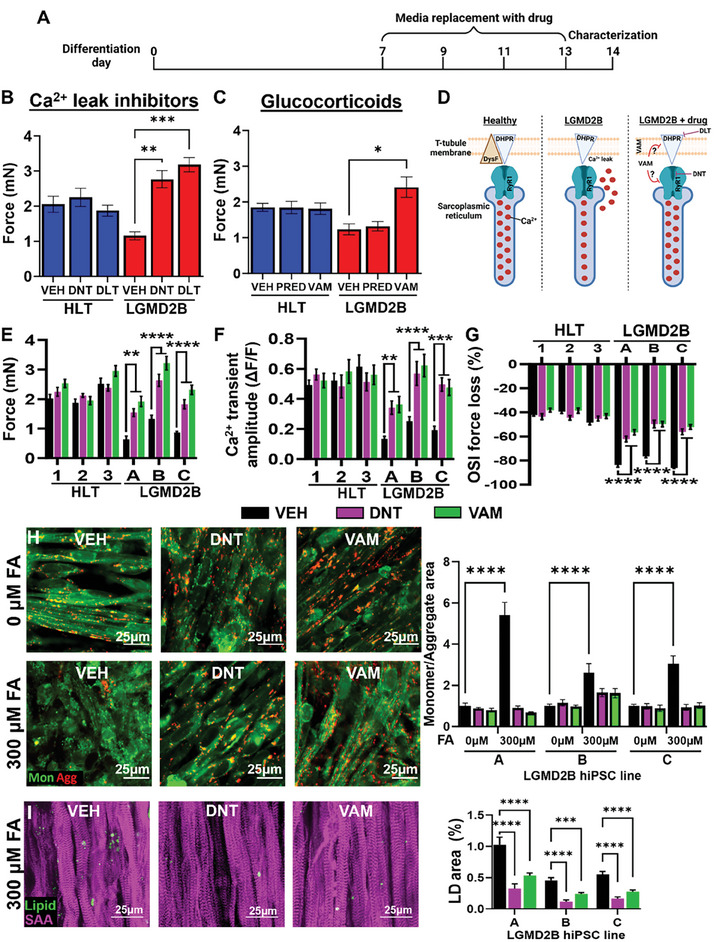
Roles of Ca^2+^ overload in LGMD2B myobundle dysfunction. A) Schematic of drug testing timeline. B,C) Quantification of force generation in healthy (HLT) and LGMD2B myobundles treated with vehicle (VEH) or B) L‐type Ca^2+^ channel inhibitor 1 µm diltiazem (DLT) or RyR inhibitor 1 µm dantrolene (DNT), or C) glucocorticoids 1 µm prednisone (PRED) or 1 µm vamorolone (VAM). D) Schematic showing expected effects of drug therapy on Ca^2+^ overload in LGMD2B muscle. E–G) Quantification of E) force generation, F) Ca^2+^ transient amplitudes (ΔF/F of Fluo‐8), and G) OSI force changes in HLT and LGMD2B myobundles treated with 1 µm DNT and 1 µm VAM (n = 4‐8 per group). H) Representative images of JC‐10 staining in LGMD2B myobundles and corresponding quantification of the ratio of green monomer (mon) to red aggregate (agg) area (n = 12 per group). I) Representative staining of LipidSpot (Lipid) and SAA and quantification of lipid droplet (LD) area (n = 12 per group). ^*^ *P* < 0.05, ^**^ *P* < 0.01, ^***^ *P* < 0.001, ^****^ *P* < 0.0001 vs. VEH. Data: mean ± SEM.

We next sought to determine mechanisms of the impaired contractile function in LGMD2B myobundles. In affected muscles of dysferlin‐deficient mice, decreased Ca^2+^ transient amplitude and increased OSI susceptibility are linked to Ca^2+^ leak into the triad junctional cleft which can be countered with the RyR1 blocker dantrolene (DNT) or the L‐Type calcium channel blocker diltiazem (DLT).^[^ ^10^, ^12^ ^]^ Alternatively, membrane repair and contractile function can be improved using the novel glucocorticoid vamorolone (VAM) but not the traditional glucocorticoid prednisolone (PRED).^[^ ^8^ ^]^ In agreement with these observations, using one healthy and one LGMD2B line, we found that one‐week treatment (**Figure** **5**A) with DNT, DLT, and VAM, but not PRED, increased contractile function in LGMD2B myobundles (Figure 5B,C). Importantly, this drug treatment did not impact healthy myobundle function, suggesting specific targeting of Ca^2+^‐handling‐dependent mechanisms of LGMD2B pathogenesis (Figure 5D). We then treated all 3 LGMD2B lines with DNT and VAM and found consistent force restoration to healthy levels without effects on the HLT lines (Figure 5E). The DNT and VAM treatment also restored Ca^2+^ transient amplitude (Figure 5F) and significantly improved functional recovery (Figure 5G) and sarcomeric structure (Figure S9, Supporting Information) following OSI injury. While DNT and VAM can improve LGMD2B muscle function in mice,^[^ ^8^, ^10^ ^]^ their effects on LGMD2B mitochondrial function and LD accumulation are unknown. We, therefore, applied VAM and DNT to FA‐treated LGMD2B myobundles and found a slight trend for increased OCR (Figure S10A–E, Supporting Information), which, however, was accompanied by a significant increase in glycolysis and decrease in OCR:ECAR ratio (Figure S10F, Supporting Information). Moreover, while DNT and VAM treatments of LGMD2B myobundles did not impact JC‐10 respiration without FAs (Figure 5H), they fully prevented mitochondrial dysfunction and reduced LD accumulation in the presence of FAs (Figure 5H,I). Together, these results indicate that DNT and VAM treatments of LGMD2B myobundles can not only rescue deficits in force generation and membrane repair but also decrease FA‐induced mitochondrial dysfunction and LD buildup. More generally, the described studies support the utility of the myobundle system as a preclinical drug screening platform to identify compounds that would restore contractile and metabolic function of dysferlin‐deficient muscle.

### 2.6 Pharmacological Induction of RyR Leak in HLT Myobundles Phenocopies the LGMD2B Phenotype

**Figure 6:**
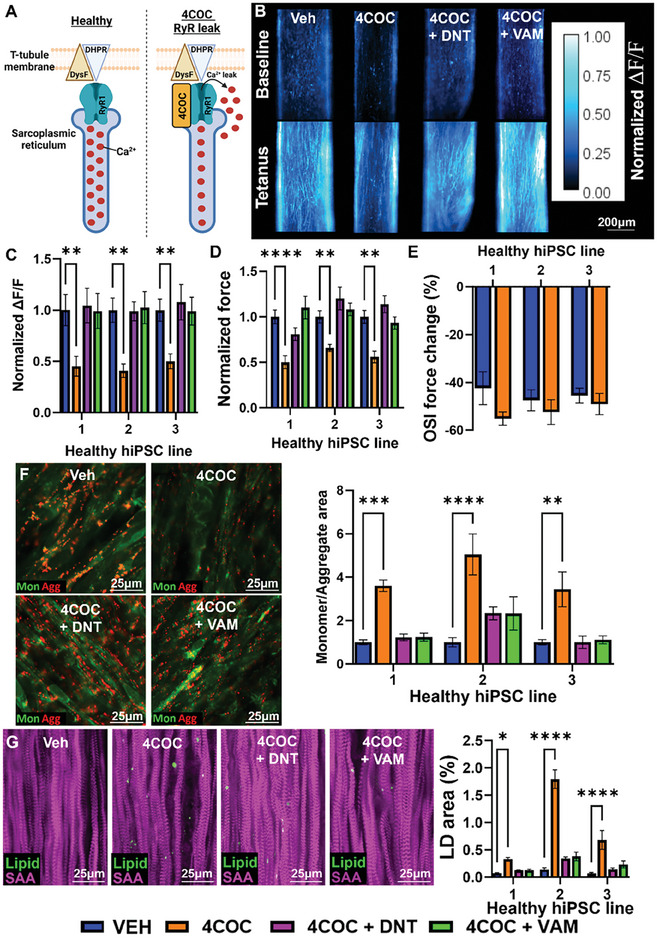
Functional and metabolic responses to pharmacologically induced RyR leak in healthy myobundles. A) Schematic depicting 4COC‐induced RyR1 Ca^2+^ leak. B,C) Representative (B) Fluo‐8 AM fluorescence intensity at baseline and peak tetanic contraction and (C) quantified Ca^2+^ transient amplitudes (ΔF/F of Fluo‐8) in vehicle (VEH), 0.5 µm 4COC, 0.5 µm 4COC + 1 µm DNT, or 0.5 µm 4COC + 1 µm VAM treated healthy myobundles (n = 12 per group). D,E) Quantified (D) tetanic force generation (n = 12 per group) and (E) OSI force recovery (n = 4‐5 per group). F) Representative images of JC‐10 staining in healthy and LGMD2B myobundles cultured in 0 or 300 µm FA and corresponding quantification of the ratio of green monomer (mon) to red aggregate (agg) area (n = 12 per group). G) Representative immunostaining of LipidSpot (Lipid) and SAA and quantification of percentage lipid droplet (LD) area (n = 12 per group). ^*^ *P* < 0.05, ^**^ *P* < 0.01, ^***^ *P* < 0.001, ^****^ *P* < 0.0001 vs. VEH. Data: mean ± SEM.

While inhibiting the RyR Ca^2+^ release with DNT prevented the pathological phenotype in LGMD2B myobundles, it remained unclear if the intracellular Ca^2+^ overload was sufficient to induce these pathological features regardless of the dysferlin deficiency. To answer this question, we induced RyR Ca^2+^ leak in healthy myobundles by treating them with the highly selective RyR activator 4‐chloro‐orto‐cresol (4COC)^[^ ^50^ ^]^ during the second week of culture (**Figure** **6**A). 4COC decreased force generation in a dose‐dependent manner, with 0.5 µm concentration yielding a 50% force reduction and no sarcomere defects (Figure S11A,B, Supporting Information). We then treated myobundles from all 3 healthy lines with 0.5 µm 4COC, and similar as in LGMD2B myobundles, found a ≈50%‐decrease in Ca^2+^ transient and force amplitude, which was reversed by co‐treatment with DNT or VAM (Figure 6B–D). On the other hand, 4COC had no adverse effects on myobundle recovery from OSI injury (Figure 6E), without or with DNT and VAM treatment (Figure S11C, Supporting Information). Finally, in response to FAs, 4COC‐treated myobundle showed impaired mitochondrial function (Figure 6F) and increased LD accumulation (Figure 6G), which were attenuated by co‐treatment with DNT or VAM. Together, these studies show that chronic RyR leak in healthy myobundles phenocopies LGMD2B‐induced loss of contractile function, LD accumulation, and mitochondrial dysfunction without impacting muscle response to OSI injury, which is independently regulated by pleotropic functions of dysferlin.

## 3 Discussion

In this study, we developed and characterized the first 3D skeletal muscle model of LGMD2B. Compared to traditional 2D culture systems, 3D tissue‐engineered muscle models permit longer culture duration, increased tissue maturation, and measurements of contractile function.^[^ ^30^ ^]^ In agreement with preclinical and clinical findings, we demonstrate that LGMD2B myobundles display reduced force generation,^[^ ^8^ ^]^ decreased Ca^2+^ transient amplitude,^[^ ^10^, ^12^ ^]^ impaired membrane repair,^[^ ^8^, ^9^, ^10^, ^12^ ^]^ and intramuscular LD accumulation.^[^ ^5^, ^51^ ^]^ By transcriptomic and metabolic analyses, we further show that the lack of dysferlin induces deficits in mitochondrial function, oxidative phosphorylation, and lipid processing. Additionally, our targeted pharmacological studies reveal that impaired force generation, mitochondrial dysfunction, and LD accumulation in LGMD2B are likely a secondary consequence to dysferlin‐deficiency‐induced Ca^[2+]^ overload, while impaired membrane repair is directly caused by the lack of dysferlin.^[^ ^9^, ^52^ ^]^ Finally, we also replicate some mouse preclinical drug responses, establishing human LGMD2B myobundles as a novel in vitro platform to promote the understanding and treatment of dysferlinopathy.

In agreement with normal muscle formation seen in patients and mice before disease onset,^[^ ^3^, ^53^ ^]^ we did not observe impaired myogenesis of iMPCs derived from any of the 3 LGMD2B hiPSC lines (Figure 1; Figure S2, Supporting Information). However, studies with primary LGMD2B MPCs derived from affected mouse^[^ ^28^, ^29^ ^]^ and human^[^ ^24^, ^25^ ^]^ muscles have shown reduced muscle fusion and MyoG levels, which was replicated by dysferlin knockdown in healthy cells.^[^ ^25^ ^]^ In contrast, we did not observe decreased MyoG levels in LGMD2B myotubes by immunostaining (Figure S2, Supporting Information) or RNA‐seq (data not shown). This discrepancy may result from methodological differences, as growth media in this study was supplemented with dexamethasone which has been shown to reduce dysferlin‐deficient fusion deficit.^[^ ^54^ ^]^ Additionally, previous studies utilized serum‐containing differentiation media,^[^ ^24^, ^25^, ^28^, ^29^ ^]^ while serum‐free differentiation media was used in the current study (Figure S3B, Supporting Information). Alternatively, hiPSC iMPCs may more accurately model fetal myogenesis^[^ ^55^ ^]^ for which dysferlin may not be required and would warrant further studies.

While muscles of dysferlin‐deficient mice show no‐to‐mild contractile deficit,^[^ ^15^, ^18^, ^19^, ^20^ ^]^ we find a robust and reproducible twofold force decrease in 2 wk LGMD2B or 4COC‐treated healthy myobundles, despite no changes in muscle architecture. This loss in contractile strength correlated with the twofold decrease in Ca^2+^ transient amplitude, implicating impaired Ca^2+^ handling (i.e. intracellular Ca^2+^ overload) as the primary source of contractile dysfunction in LGMD2B myobundles. In mouse LGMD2B myofibers, the lack of dysferlin results in a ≈15% decrease in Ca^2+^ transient amplitude,^[^ ^10^, ^56^ ^]^ suggesting that LGMD2B mouse myofibers exhibit less Ca^2+^ overload compared to human LGMD2B myobundles. This difference may stem from species‐specific differences in SR Ca^2+^ leak propensity,^[^ ^57^ ^]^ and/or developmentally immature triad structure,^[^ ^35^ ^]^ or Ca^2+^ sequestering capacity of myobundles. Still, similar to LGMD2B mouse studies,^[^ ^10^, ^12^ ^]^ we find that reducing Ca^2+^ load with the LTCC inhibitor diltiazem or RyR inhibitor dantrolene restores contractile function and membrane repair capacity of LGMD2B myobundles. On the other hand, our 4COC studies show that increased SR Ca^2+^ leak alone is insufficient to impair the membrane repair capacity of healthy muscle (Figure 6E). Thus, loss of other dysferlin functions, such as vesicle trafficking and fusion at sites of membrane damage, rather than Ca^2+^ overload, are a likely cause for impaired membrane repair of LGMD2B muscle.^[^ ^43^, ^52^ ^]^

Similar to studies in LGMD2B mice,^[^ ^8^ ^]^ we also found that the novel glucocorticoid vamorolone (VAM), unlike traditionally used prednisolone, restored force generation in LGMD2B myobundles (Figure 5C), in addition to recovering Ca^2+^ transient amplitude and membrane repair (Figure 5E–G). VAM has shown similar therapeutic efficacy to traditional glucocorticoids with an increased safety profile, and recently gained FDA approval for use in Duchenne muscular dystrophy (DMD) patients.^[^ ^58^ ^]^ In DMD and LGMD2B cells, VAM but not PRED decreases membrane lipid fluidity resulting in increased sarcolemmal protection and repair capacity.^[^ ^8^ ^]^ Interestingly, in smooth muscle, increased membrane fluidity results in Ca^2+^ overload, which can be prevented by the LTCC inhibitor diltiazem.^[^ ^59^ ^]^ This suggests that VAM may stabilize sarcolemma in LGMD2B muscle and in turn decrease Ca^2+^ overload with benefits similar to specific Ca^2+^ channel (LTCC or RyR) inhibitors. However, VAM also restored Ca^2+^ transients with 4COC‐induced RyR leak in healthy myobundles, where membrane fluidity is likely unchanged as membrane repair capacity was unaltered. While the precise mechanisms of VAM action on LGMD2B muscle require further characterization, our studies show that diverse drug classes can mitigate LGMD2B pathology and support the utility of LGMD2B myobundles as a drug screening platform.

Increased cytosolic Ca^2+^ load in muscle cells requires increased extrusion by Ca^2+^ pumps which preferentially utilize glycolysis to sustain activity,^[^ ^60^, ^61^ ^]^ which may in part explain the increased glycolysis in LGMD2B myobundles. However, compensatory increase in glycolysis also occurs with mitochondrial dysfunction to meet cellular energy demands.^[^ ^62^ ^]^ At the genetic level, mitochondrial dysfunction was indicated from transcriptomic analysis of LGMD2B myobundles (Figure 3D,E) and native muscle (Figures S4–S6, Supporting Information). Functionally, both LGMD2B myobundles and 4COC‐treated healthy myobundles displayed multiple features of mitochondrial dysfunction only when supplemented with FAs, including: (1) inability to increase oxidative metabolism, (2) decreased mitochondrial membrane potential, and (3) LD accumulation (Figure 4). For the first time, we showed that DNT and VAM partially reversed these metabolic deficiencies (Figure 5C–I) suggesting that intracellular Ca^2+^ overload significantly contributes to mitochondrial dysfunction and prevents metabolic adaptations to FA supplementation in LGMD2B. However, dysferlin may also directly contribute to mitochondrial dysfunction as DNT and VAM treatments of LGMD2B myobundles did not rescue OCR increase in response to FAs (Figure S9A–E, Supporting Information) and they even further increased glycolytic metabolism (Figure S9F, Supporting Information). The direct effects of dysferlin on mitochondrial function could be mediated via formed complexes with inner and outer mitochondrial membrane proteins^[^ ^45^ ^]^ or a splice variant that contains a mitochondrial localization signal.^[^ ^63^ ^]^ Overall, our results in myobundles are consistent with reports that LGMD2B patient myofibers show structural mitochondrial abnormalities^[^ ^64^, ^65^ ^]^ and decreased protein levels and activities of mitochondrial complexes I and IV.^[^ ^66^, ^67^ ^]^ As mitochondria also play important roles in membrane repair^[^ ^68^, ^69^ ^]^ and calcium homeostasis,^[^ ^70^, ^71^ ^]^ the specific roles of mitochondrial dysfunction in LGMD2B pathology warrant further investigation.

Intramyocellular LD accumulation has been observed in LGMD2B mouse and patient muscle,^[^ ^5^ ^]^ but if this occurs due to muscle cell‐autonomous mechanisms, cellular crosstalk, altered systemic factors, or the pro‐inflammatory LGMD2B environment remains unclear. To our knowledge, our study is the first to demonstrate intracellular LD accumulation in LGMD2B muscle without the confounding effects of the in vivo environment or the presence of additional cell types. LDs store lipids for fatty acid oxidation, membrane synthesis, and lipid signaling precursors while minimizing lipotoxicity.^[^ ^72^ ^]^ In this study, LD accumulation appears to predominantly be a consequence of perturbed Ca^2+^ homeostasis, as inducing RyR leak in healthy myobundles increased LD accumulation and restoring Ca^2+^ transient amplitude with DNT or VAM significantly reduced LD accumulation. In agreement with our findings, LD accumulation has been observed in a subset of patients with malignant hyperthermia and early‐onset hypotonia where RyR mutations cause pathogenic Ca^2+^ leaks.^[^ ^73^, ^74^ ^]^ However, greater lipid‐handling abnormalities are seen in patients with LGMD2B compared to RyR myopathies. Thus, a Ca^2+^‐independent role for dysferlin in regulating LD biogenesis, localization, or metabolism cannot be ruled out. In fact, dysferlin binds to LDs and is required for their correct cellular localization in cardiomyocytes,^[^ ^75^ ^]^ and dysferlin deficiency promotes FA storage in pre‐symptomatic mouse myofibers.^[^ ^51^ ^]^ Moreover, mitochondrial dysfunction, which we find in FA‐supplemented LGMD2B myobundles, also induces LD accumulation.^[^ ^76^ ^]^ Additionally, dysferlin deficiency impacts the muscle lipidome^[^ ^51^ ^]^ and disease severity is exacerbated by increased circulating cholesterol levels.^[^ ^77^, ^78^ ^]^ The serum‐free myobundle culture allows controlled supplementation of extracellular FAs and cholesterol, thus providing a platform to dissect the precise roles of dysferlin in lipid and mitochondrial metabolism.

Dysferlinopathies represent an ideal myopathy to model using hiPSC technology due to the multi‐functional roles of dysferlin, multicellular involvement, mild disease severity in mouse models, and paucity of primary muscle biopsies. Comparisons of our RNA‐seq dataset with previously published human and mouse DNA microarray datasets showed several similarities across species and experimental models including significant downregulation of genes related to Ca^2+^ handling and muscle contraction. Furthermore, we found significant downregulation of genes related to protein synthesis, RNA maturation, and ribosome biogenesis, consistent with muscle atrophy and atrogene upregulation found in LGMD2B patients.^[^ ^79^ ^]^ Interestingly, we also found differential regulation of neuronal projection and neuromuscular junction assembly processes, which may suggest involvement of post‐synaptic dysfunction in LGMD2B.^[^ ^80^ ^]^ Additionally, genes related to smoothened signaling and cilia formation were upregulated in LGMD2B myobundles, which could reflect altered lipid‐handling.^[^ ^81^ ^]^ The biggest discrepancy between transcriptomes of the unicellular LGMD2B myobundles and native LGMD2B muscle was the lack of immune cell markers. Thus, future advances to the LGMD2B myobundle model will involve the incorporation of immune^[^ ^15^, ^16^ ^]^ and fibroadipogenic progenitor^[^ ^14^ ^]^ cells to model and study the mechanisms leading to extensive immune cell infiltration and ectopic fat formation in LGMD2B patients.

## 4 Conclusion

In summary, we have developed the first 3D tissue‐engineered model of LGMD2B. This model displays: (1) significant contractile weakness not observed in traditional mouse models, (2) deficits in Ca^2+^ handling and membrane repair, (3) impaired mitochondrial adaptations to FA challenge, and (4) pharmacological responses seen in mouse models. As such, the human LGMD2B myobundle model is expected to complement transgenic mice as a novel preclinical platform for mechanistic studies and therapeutic testing for dysferlinopathies.

## 5 Experimental Section

### Myogenic differentiation of hiPSCs into iMPCs: Differentiation of hiPSCs to iMPCs

Human induced pluripotent stem cell (hiPSC) lines were differentiated into iMPCs as previously described.^[^ ^35^ ^]^ CGWT (kindly gifted by Charles Gersbach) and JFWT, JFHZ, JFRBi, JFNY, JFMD (WiCell) hiPSC lines were maintained in feeder‐free conditions in E8 or mTESR plus medium (Stemcell Technologies) and were routinely tested for Mycoplasma contamination using commercially available kits (MycoAlert, Lonza). No differences in cell morphology, proliferation rate, or ability to generate iMPCs were detected among the 6 lines. hiPSC colonies were dissociated into single cells with Accutase (Stemcell Technologies) and seeded onto Matrigel (Corning) coated 6‐well plates at a cell density of 1 × 10^3^ cm^−2^. 24 h after plating, cells were transduced with a lentivirus conferring Doxycycline (Dox)‐inducible Pax7 and GFP expression.^[^ ^35^ ^]^ The transduced hiPSCs were expanded in E8 media for 3–20 passages, then dissociated into single cells with Accutase, and seeded onto Matrigel‐coated 6‐well plates in E8 or mTESR plus supplemented with Y27632 (5 µm, Tocris) at 3.3 × 10^4^ cells cm^−2^. The following day, Y27632 was removed, and cells were cultured for 2 days in the presence of CHIR99021 (10 µm in E8 or mTESR plus media, Selleck Chemical), followed by 18‐day culture in E6 media supplemented with 1 µg mL^−1^ Dox (Sigma) to induce myogenic differentiation. At the end of the 18‐day period, Pax7+/GFP+ induced myogenic progenitor cells (iMPCs) were purified by fluorescence‐activated cell sorting for GFP (FACS, see below). During differentiation of iMPCs, 10 ng mL^−1^ bFGF (R&D) was added starting at day 5 to enhance the proliferation of GFP+ cells.

### Fluorescent‐Activated Cell Sorting of iMPCs

On differentiation day 19–22, cells were dissociated with 0.25% Trypsin‐EDTA (Thermo) and washed in neutralizing media (Table S1, Supporting Information). Detached cells were centrifuged at 300 g for 5 min, then resuspended in a sorting solution (Table S1, Supporting Information) and filtered through 30 µm filter (SYSMEX) to remove cell clusters and debris. Single‐cell suspensions were kept on ice until sorting, with undifferentiated hiPSCs used as negative control. Cells were sorted for GFP using MoFlo Astrios cell sorter (Beckman Coulter) in Duke University Flow Cytometry Shared Resource.

### Expansion of iMPCs

After sorting, GFP+ iMPCs were kept on ice in collecting solution (Table S1, Supporting Information), centrifuged at 300 g for 5 min, and resuspended in fresh E6 media supplemented with Y27632, Dox, and bFGF, then seeded at 4 × 10^4^ cm^−2^ in Matrigel‐coated flasks. 24–48 h post‐sorting, cells were incubated in expansion media (EM, Table S1, Supporting Information), supplemented with Dox and bFGF, and passaged at a 1:3–1:6 ratio after reaching 80% confluence.

### 2D Differentiation of iMPCs

iMPCs were seeded at the density of 1 × 10^5^ cm^−2^ on Matrigel‐coated dishes and after reaching 100% confluence, EM was washed out with PBS and switched to differentiation media (DM) 1 (Table S1, Supporting Information) for the first 4 days of culture and then DM 3 (Table S1, Supporting Information) for the remainder of culture. The media was changed every 2 days. For treatment with fatty acids, 300 um fatty acids (1:1:1 oleic, linoleic, and palmitic acid) were added to DM 3.

### Fabrication and Differentiation of Myobundles

3D engineered muscle tissues (myobundles) were formed within polydimethylsiloxane (PDMS) molds containing two semi‐cylindrical wells (5 mm long, 1 mm diameter), cast from 3D‐machined Teflon masters (Figure S3A, Supporting Information), similar to our previously described methods.^[^ ^35^, ^37^, ^39^, ^42^ ^]^ PDMS molds were coated with 0.2% (w/v) pluronic F127 (Invitrogen) for 1 h at room temperature to prevent hydrogel adhesion. Laser‐cut Cerex frames (7 × 7 mm^2^, 1 mm wide rim) positioned around the 2 wells served to anchor myobundle ends and facilitate handling and implantation. Cell/hydrogel mixture (Table S1, Supporting Information) was injected into the PDMS wells and polymerized at 37 °C for 30  min. Formed myobundles were kept on rocking platform in EM supplemented with 1 µg mL^−1^ Dox and 1.5 mg mL^−1^ 6‐aminocaproic acid (ACA, Sigma) for 4 days. Media was then switched to DM 1 for 4 days, followed by DM 2 for 3 days, and then DM 3 for the remainder of the culture with media changed every 2 days (Figure S3B, Supporting Information).

### Force Measurements

Contractile force generation in myobundles was assessed using a custom force measurement set‐up as previously described.^[^ ^35^, ^37^, ^39^, ^42^ ^]^ Briefly, single myobundles attached to frame were transferred to a measurement bath with DM equilibrated at 37 °C. One end of the tissue was pinned to a fixed PDMS block and the other end was pinned to a PDMS float attached to a force transducer mounted on a motorized linear actuator (Thorlabs, Newton, NJ). The sides of the frame were cut to allow isometric measurement of contractile force and stretching of the tissue by the actuator. To assess the force–length relationship, myobundle was stretched in 5% steps, and at each step, tissue was stimulated with a 40 V cm^−1^, 10 ms long electrical pulse using a pair of platinum electrodes, and the twitch force was recorded. At 20% stretch, 1s long stimulations at 5, 10, 20 40, and 60 Hz were applied, and the contractile force was recorded to assess the force–frequency relationship. Contractile force traces were analyzed for peak tetanus force using a custom MATLAB program.

### Hypo‐Osmotic Shock Injury Measurements

Osmotic shock injury was performed at the end of functional testing. Peak tetanic force was measured under a 1‐s 60 Hz electrical stimulus train in normo‐osmotic DM media. The osmotic shock was then induced by replacing normo‐osmotic media with ≈30 mOsm media (hypo‐osmotic, 90% DI water, and 10% DM) and tetanus was induced every 60 s. After 5 min, hypoosmotic media was washed out quickly 2–3 times with DM, and myobundles were kept in DM for an additional 15 min. OSI force change was calculated as the % force change at the end relative to before the OSI protocol.

### Calcium Transient Analysis

For Ca^2+^ transient measurements, myobundles were incubated with 10 µm of calcium‐sensitive dye Fluo‐8 AM (AAT Bioquest, 21080) in DM in an incubator for 1 h while rocking, followed by washing in dye‐free media for 30 min. Electrically induced Ca^2+^ transients were recorded as previously described.^[^ ^35^, ^42^ ^]^ Myobundles were transferred into a glass‐bottom live‐imaging chamber with Tyrode's solution and 10 um blebbistatin warmed at 37 °C. Fluorescence images were acquired at ×4 magnification on a Nikon microscope using a high speed EMCCD (electron multiplying charge‐coupled device) camera (Andor iXon 860) and Andor Solis software. Ca^2+^ transient amplitudes were calculated as the maximum relative change in fluorescence signal, Δ*F*/*F*  = (Peak − Trough)/(Trough − Background) using Andor Solis software.

### Immunofluorescence

Cells cultured in monolayers were fixed in 4% paraformaldehyde in PBS for 15 min at room temperature. Myobundles were fixed in 2% paraformaldehyde in PBS overnight at 4 °C while rocking. For cross‐sectional analysis, fixed samples were submerged in optimal cutting temperature compound (Electron Microscopy Sciences) and snap‐frozen in liquid nitrogen. Myobundles were sectioned (10 µm thick) using a cryostat (LEICA CM1950), and then mounted onto charged glass slides. Before staining, sections were incubated in a blocking solution containing 5% chick serum and 0.1% Triton X‐100 for 45 min. Primary antibodies were applied in the blocking solution overnight at 4 °C, followed by the application of secondary antibodies overnight at 4 °C. Antibody information and dilutions are provided in supplementary information (Table S2, Supporting Information). Immunostained samples were mounted with ProLong Glass Antifade reagent (Thermo Fisher Scientific, P36984). Fluorescence images were acquired using a Leica SP5 confocal microscope at ×20 or ×40 magnification and analyzed by ImageJ.

### RNA Sequencing Analysis

RNA sequencing analysis was performed based on our previously described methods.^[^ ^37^ ^]^ Specifically, RNA was isolated from pooled human myobundles (4 per sample) using the Bullet Blender (Next Advance) and the Aurum total RNA Mini Kit (Biorad). The samples were processed by the Duke Center for Genomic and Computational Biology. RNA‐seq data was processed using the TrimGalore toolkit which employs Cutadapt to trim low‐quality bases and Illumina sequencing adapters from the 3’ end of the reads. Only reads that were 20 nt or longer after trimming were kept for further analysis. Reads were mapped to the GRCh38v93 version of the human genome and transcriptome using the STAR RNA‐seq alignment tool. Reads were kept for subsequent analysis if they mapped to a single genomic location. Gene counts were compiled using the HTSeq tool. Only genes that had at least 10 reads in any given library were used in subsequent analysis. Normalization and differential expression were carried out using the DESeq2 Bioconductor package with the R statistical programming environment. In each analysis, parental line was included as a cofactor in the model. The false discovery rate was calculated to control for multiple hypothesis testing. Gene set enrichment analysis was performed on all genes ranked by FDR in GSEA version 4.2.2 to identify gene ontology terms and pathways associated with altered gene expression for each of the comparisons performed.

### DNA Microarray Analysis

Human (GSE109178)^[^ ^47^ ^]^ and mouse (GSE25904^[^ ^82^ ^]^ and GSE62945^[^ ^49^ ^]^) microarray datasets were obtained from the GEO databank. GSE109178 contained 6 healthy and 8 LGMD2B samples isolated from the vastus lateralis.^[^ ^47^ ^]^ Microarray datasets were analyzed using the GEOexplorer web interface^[^ ^83^ ^]^ using Benjaminii & Hochberg (false discovery rate) and limma precision weights (vooma) statistical analysis. Gene set enrichment analysis was performed on all genes ranked by FDR in GSEA (version 4.2.2) to identify gene ontology terms and pathways associated with altered gene expression for each of the comparisons performed. To allow comparisons of RNA‐seq and DNA microarray datasets, the top 100 significantly different GOBP terms were manually classified into 13 functional categories that best represented the range of significantly different biological processes across all datasets.

### Seahorse analysis: 2D Seahorse Analysis

2D seahorse analysis was performed with a Seahorse XFe96 extracellular flux analyzer by plating iMPCs at 20K/well in EM. Two days after plating, differentiation was induced with DM, and cellular bioenergetics assessed on differentiation day 8. Mitochondrial function was assessed by Mito stress test assay (Agilent) according to the manufacturer's instructions. Specifically oxygen consumption rate (OCR, pmol min^−1^) was measured upon sequential addition of the following compounds into each culture well: Oligomycin (complex V electron transport chain (ETC) inhibitor, 1 µm), carbonyl cyanide‐4(triflouromethoxy)phenylhydrazone (FCCP, uncoupling agent to induce maximal respiration rate, 1 µm), and antimycin and rotenone A (inhibit complexes I and III, respectively, to inhibit all ETC activity, 0.5 µm). The glycolytic function was measured by the Glycolysis stress test assay (Agilent) according to the manufacturer's instructions. Specifically, extracellular acidification rate (ECAR, mpH min^−1^) was measured upon sequential addition of the following compounds into each culture well: glucose (10 mm), oligomycin (1 um), and 2‐deoxy‐D‐glucose (to inhibit glycolysis, 50 mm). Measured OCR and ECAR values for individual wells were normalized per their protein contents determined by BCA assay following cell lysis in cell lysis buffer (Abcam). Assessment of relative oxidative‐to‐glycolysis rates was measured by determining the OCR:ECAR ratio.

### 3D Seahorse Analysis

3D seahorse analysis was performed with a Seahorse XFe24 extracellular flux analyzer. Miniaturized myobundles (2 mm × 1mm) containing 20 K cells per tissue were utilized since they fit into Seahorse XFe24 culture plates. These miniaturized tissues were cultured in the same manner as regular‐sized myobundles and cellular bioenergetics were assessed on differentiation day 14 by Mitostress test assay. To prevent the induction of hypoxia, an optimized previously described^[^ ^37^ ^]^ custom‐made media with physiological amino acid levels was used. Optimized concentrations of Oligomycin (2.5 µm), FCCP (2 µm), and antimycin and rotenone A (0.5 µm) were sequentially injected into culture wells. Measured OCR and ECAR values for individual wells were normalized per their protein contents determined by BCA assay following cell lysis in cell lysis buffer (Abcam). Assessment of relative oxidative‐to‐glycolysis rates was measured by determining the OCR:ECAR ratio.

### JC‐10 Assay

Mitochondrial membrane potential was assessed by a JC‐10 assay (AAT Bioquest, 22204) per the manufacturer's instructions. Specifically, 2‐wk miniaturized myobundles were incubated with 30 µm JC‐10 for 30 min in DM. Myobundles were then washed with PBS and imaged live using a Leica SP5 confocal microscope by 488 nm laser excitation. Green fluorescence representing the monomeric form of JC‐10 was detected by emission at 525–540 nm. Red emission representing a potential‐dependent aggregation of JC‐10 in the mitochondria was detected by emission at 590–605 nm.

### Live/Dead Assay

Cell viability was assessed by a LIVE/DEAD^TM^ assay (ThermoFisher, L3224) per manufacturer's instructions. Specifically, miniaturized myobundles were incubated with 2 µm calcein green AM and 4 µm ethidium homodimer‐1 for 15 min in PBS at room temperature to label live and dead cells, respectively. Green fluorescence representing live cells was detected by excitation at 488 nm and emission at 525–540 nm. Red emission representing dead cells was detected by excitation at 561 nm and emission at 605–645 nm. Dead positive controls were generated by incubating myobundles in 70% ethanol at room temperature for 15 min.

### Statistical Analysis

Experimental data are reported as mean ± SEM. Statistical significances were evaluated by nested *t*‐test when comparing all 3 HLT to all 3 LGMD2B lines. One‐way or two‐way ANOVA with Tukey–Kramer HSD tests were used when comparing effects of drug treatments to vehicle controls using GraphPad Prism software. P−value < 0.05 was considered statistically significant. Sample sizes for experiments were determined based on variance of previously reported measurements.

## Conflict of Interest

The authors declare no conflict of interest.

## Author Contributions

A.K. and N.B. performed conceptualization. A.K., N.K.P., M.D.K., G.T., T.K., and N.B. performed methodology. A.K., N.K.P., T.R., M.B., A.D., T.R., and Z.D.F. performed investigation. A.K., N.K.P., M.B., A.D., and Z.D.F. performed visualization. G.T., T.K., and N.B. performed supervision. A.K. Wrote—original draft. A.K., N.K.P., T.R., M.D., A.D., Z.D.F., M.E.K., G.A.T., T.K., and N.B. wrote, reviewed and edited the manuscript.

## Supporting information

Supporting Information

Supplemental Movie 1

## Data Availability

The data that support the findings of this study are available from the corresponding author upon reasonable request.
