# Supplementary material for: Bioengineered Model of Human LGMD2B Skeletal Muscle Reveals Roles of Intracellular Calcium Overload in Contractile and Metabolic Dysfunction in Dysferlinopathy
Source: Adv Sci (Weinh). 2024 Jun 17;11(31):2400188. doi: 10.1002/advs.202400188 (PMC11336985; doi:10.1002/advs.202400188)
Supplement: Supplementary file 1 — Supporting Information [file ADVS-11-2400188-s001.pdf]

ADVANCED SCIENCE
Open Access

Supporting Information

for *Adv. Sci.*, DOI 10.1002/advs.202400188

Bioengineered Model of Human LGMD2B Skeletal Muscle Reveals Roles of Intracellular Calcium Overload in Contractile and Metabolic Dysfunction in Dysferlinopathy

Alastair Khodabukus, Neel K. Prabhu, Taylor Roberts, Meghan Buldo, Amber Detwiler, Zachary D. Fralish, Megan E. Kondash, George A. Truskey, Timothy R. Koves and Nenad Bursac**

Supporting Information

Title Bioengineered model of human LGMD2B skeletal muscle reveals roles of intracellular calcium overload in contractile and metabolic dysfunction in dysferlinopathy.

Authors
Alastair Khodabukus,[1*] Neel K. Prabhu,[1] Taylor Roberts,[1] Meghan Buldo,[1] Amber DeTwiler,[1] Zachary D.Fralish,[1] Megan E. Kondash,[1] George A. Truskey,[1] Tim Koves,[2] Nenad Bursac[1*]

*Author(s), and Corresponding Author(s)**

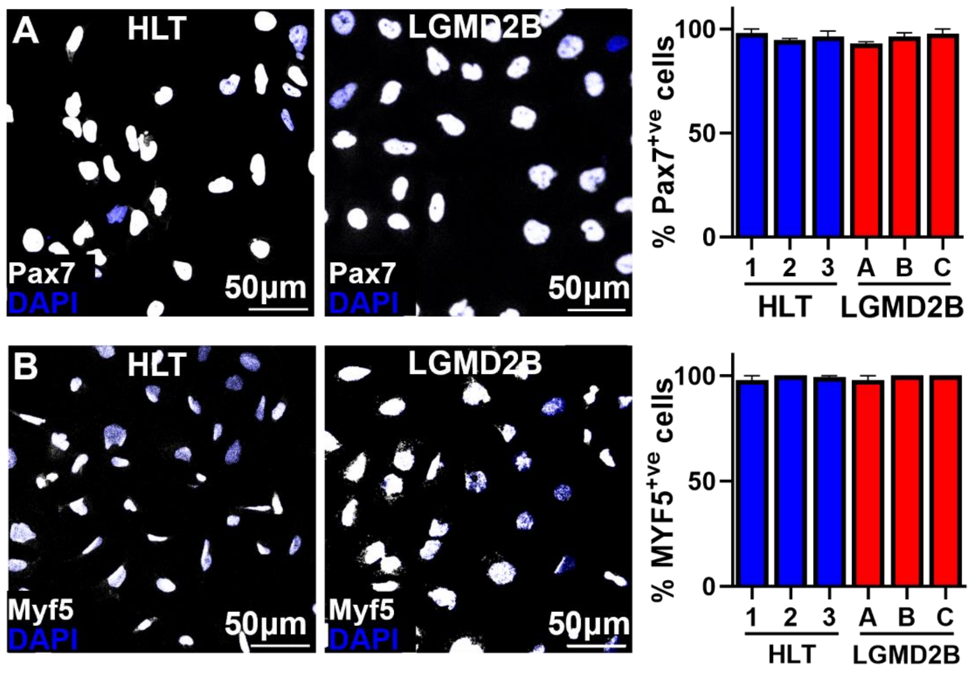

Figure S1. Myogenic regulatory factor expression in healthy and LGMD2B iMPCs. Representative images and quantifications of passage 2 iMPCs stained for (**A**) Pax7 and (**B**) Myf5 (N = 1 differentiation and n = 3 replicates per hiPSC line).

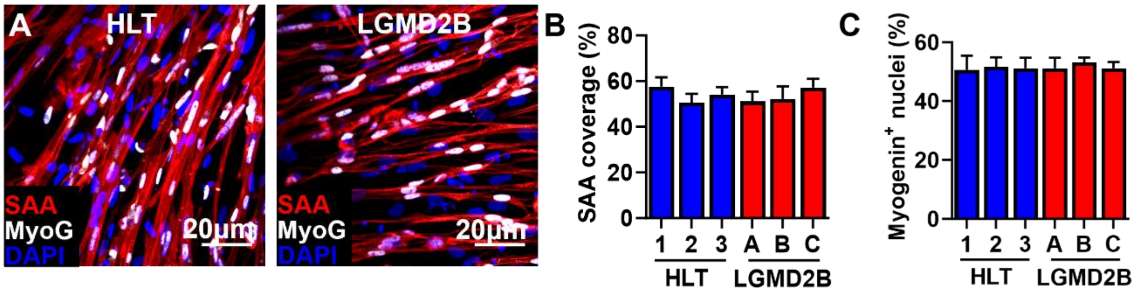

Figure S2. Two-dimensional myogenic differentiation of healthy and LGMD2B iMPCs. Healthy and LGMD2B iMPCs were differentiated for 8 days in traditional two-dimensional culture. (**A**) Representative images of cultures stained for sarcomeric alpha-actinin (SAA),

myogenin (MyoG), and DAPI. (**B**-**C**) Quantifications of (**B**) SAA area and (**C**) MyoG-positive nuclei (N = 1 differentiation and n = 3 replicates per hiPSC line).

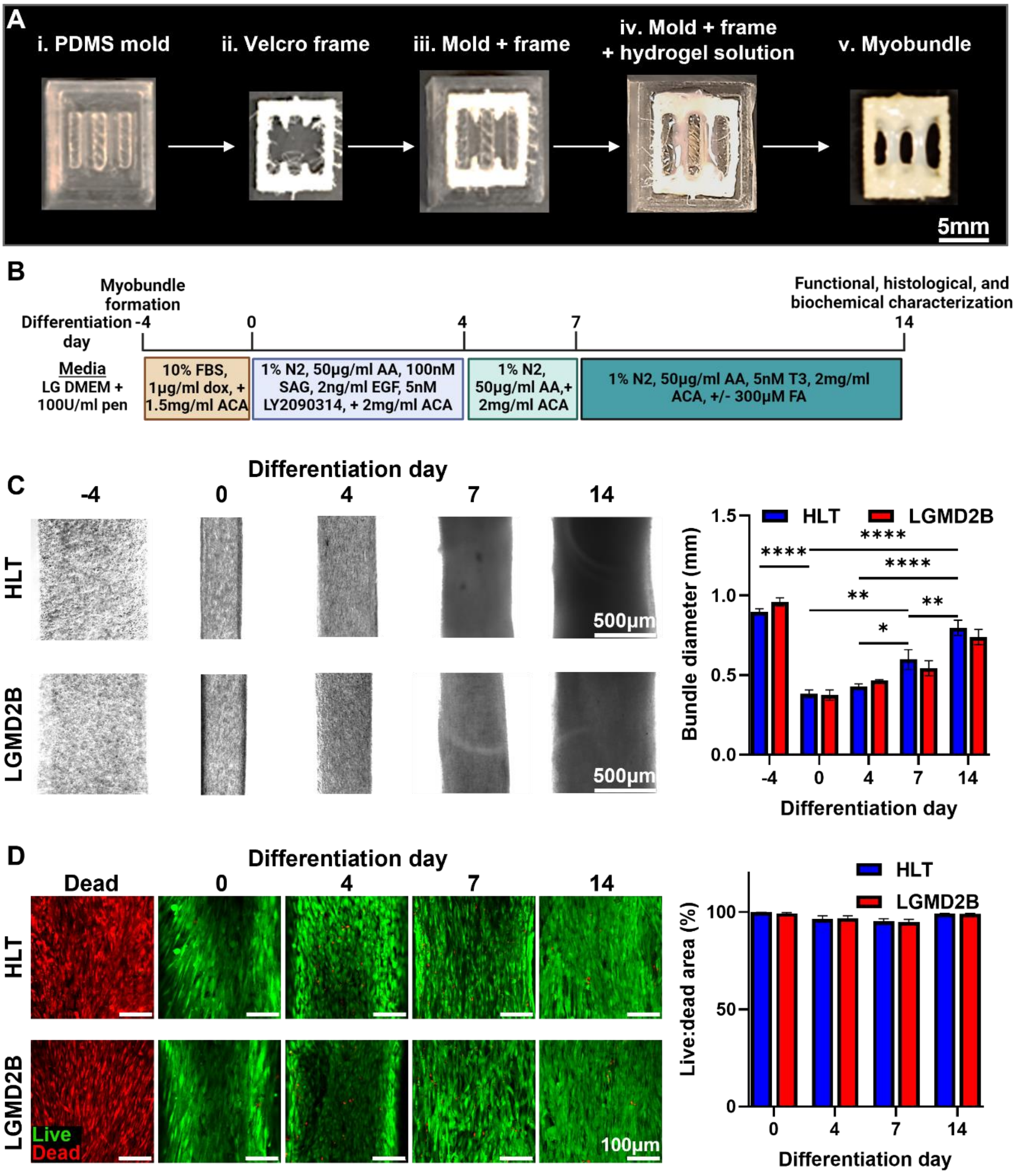

Figure S3. Myobundle formation and culture. (**A**) Representative images of myobundle fabrication process. (**B**) Schematic depicting media composition used during myobundle culture. (**C**) Representative brightfield images and quantification of myobundle width over culture duration (N = 1 differentiation and 1 hiPSC line, n = 7-8 replicates per line) (**D**) Representative live/dead images and corresponding quantification (N = 1 & 1 hiPSC line, n = 6). LG, low glucose; pen, penicillin; dox, doxycycline; FBS, fetal bovine serum; ACA, aminocaproic acid; AA, ascorbic acid-2-phosphate; SAG, smoothened agonist; EGF, epidermal growth factor; LY2090314, GSK3β inhibitor; T3, triiodothyronine; FA, fatty acid. *P<0.05, **P<0.01, ****P<0.0001

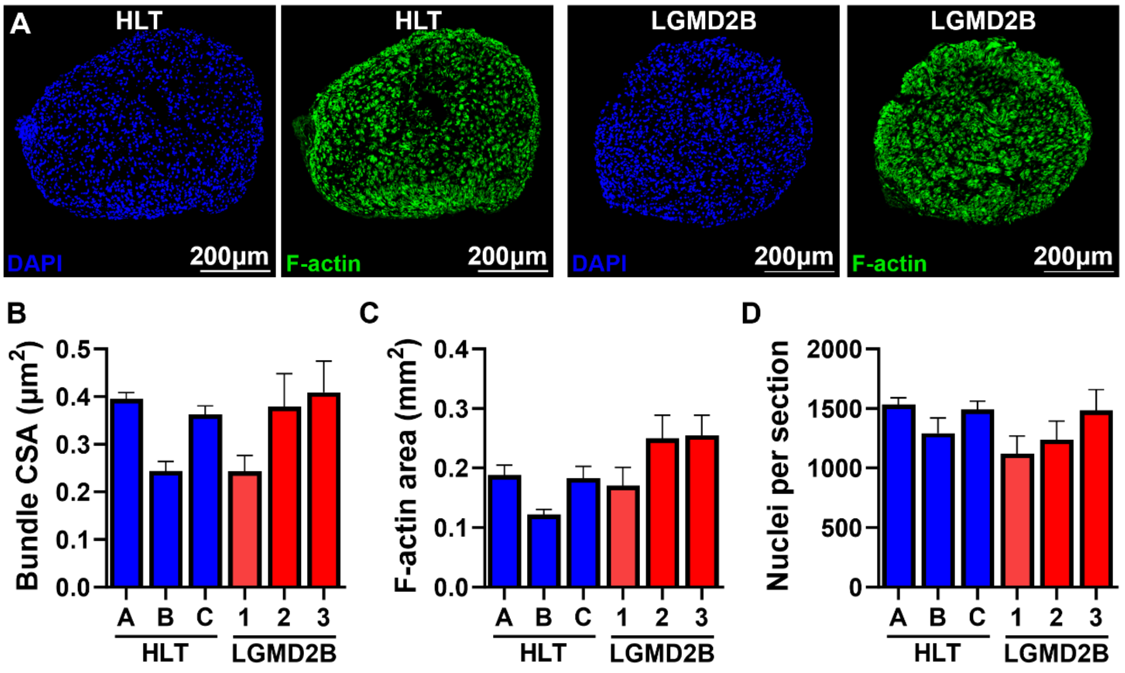

Figure S4. Cross-sectional analysis of 2-wk healthy and LGMD2B myobundles. (**A**) Representative images of 2-wk myobundle cross-sections stained for filamentous actin (F-actin) and DAPI. (**B-C**) Quantifications of (**B**) entire myobundle cross-sectional area (CSA), (**C**) F-actin^{+} area, and (**D**) nuclei per cross-section (N = 1 differentiation and n = 6-8 myobundles per hiPSC line).

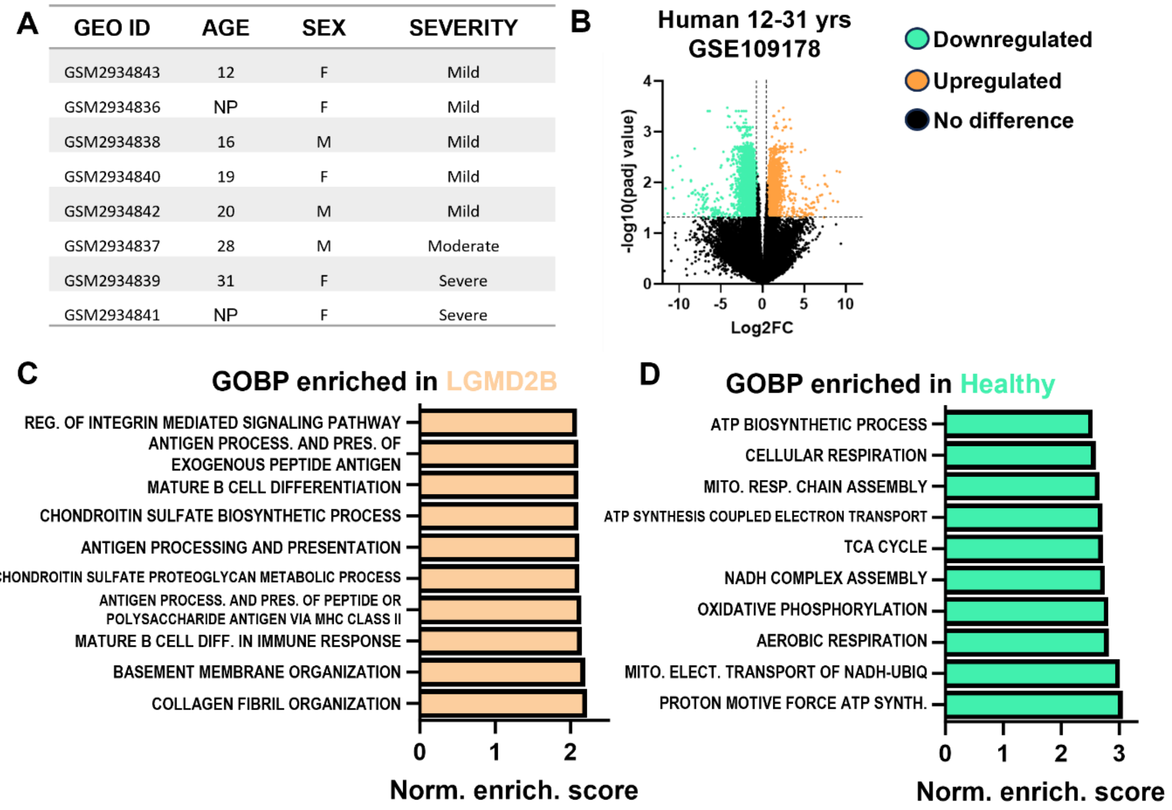

GEO ID	AGE	SEX	SEVERITY
GSM2934843	12	F	Mild
GSM2934836	NP	F	Mild
GSM2934838	16	M	Mild
GSM2934840	19	F	Mild
GSM2934842	20	M	Mild
GSM2934837	28	M	Moderate
GSM2934839	31	F	Severe
GSM2934841	NP	F	Severe

Figure S5. Microarray analysis of human LGMD2B muscle samples. (**A**) LGMD2B patient information (NP = not provided). (**B**) Volcano plot of probes detected in GSE109178. (**C-D**) Top 10 differentially regulated gene ontology biological processes (GOBP) in (**C**) LGMD2B and (**D**) healthy donors.

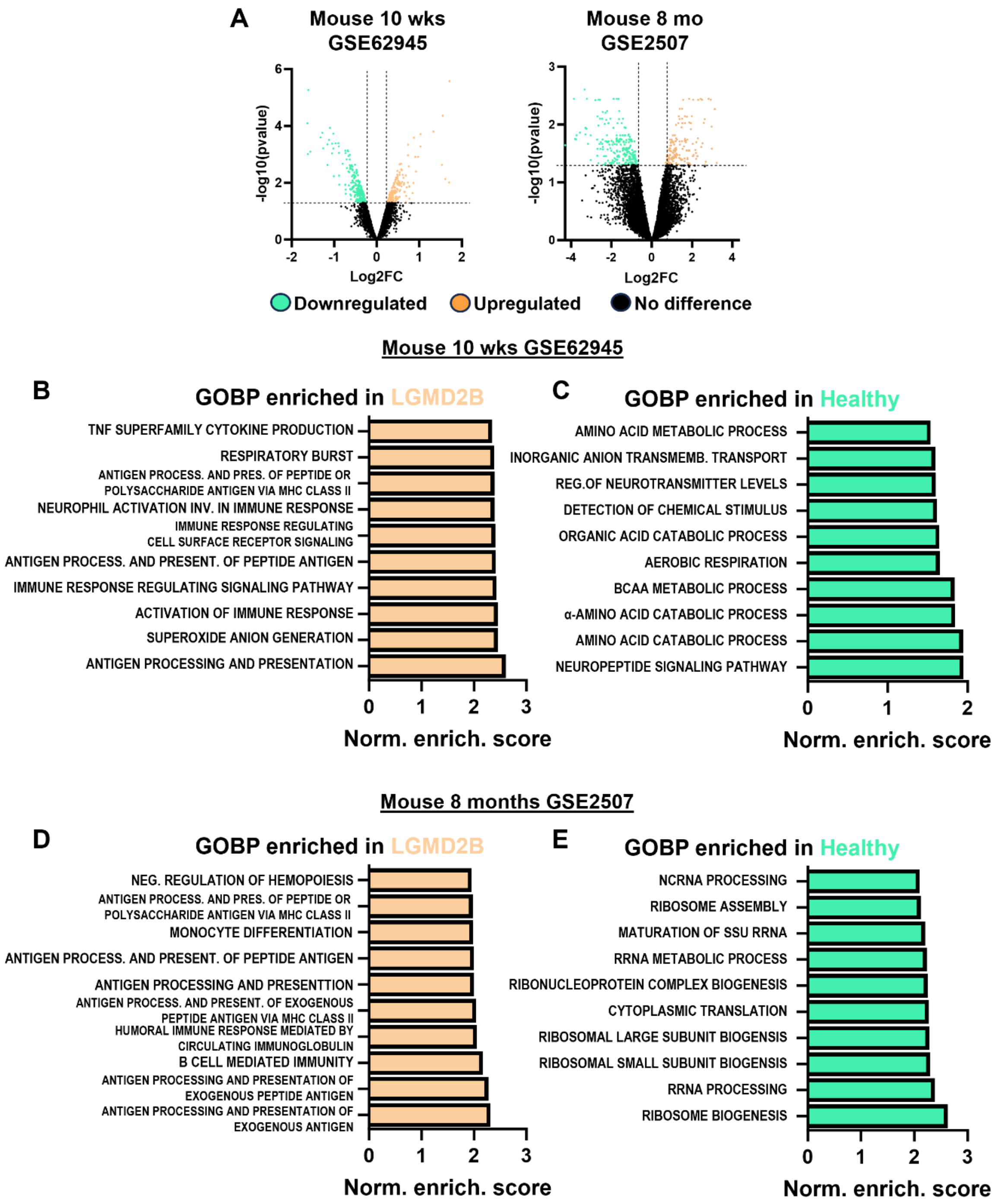

Figure S6. Microarray analysis of presymptomatic and symptomatic mouse LGMD2B muscle samples. (**A**) Volcano plots of probes detected in healthy and presymptomatic (10 weeks, GSE62945) and symptomatic (8 months, GSE2507) LGMD2B mouse muscle. (**B-E**) Top 10 differentially regulated gene ontology biological processes (GOBP) in (**B**) presymptomatic LGMD2B and (**C**) healthy 10-week mice, and (**D**) symptomatic LGMD2B and (**E**) healthy 8-month mice.

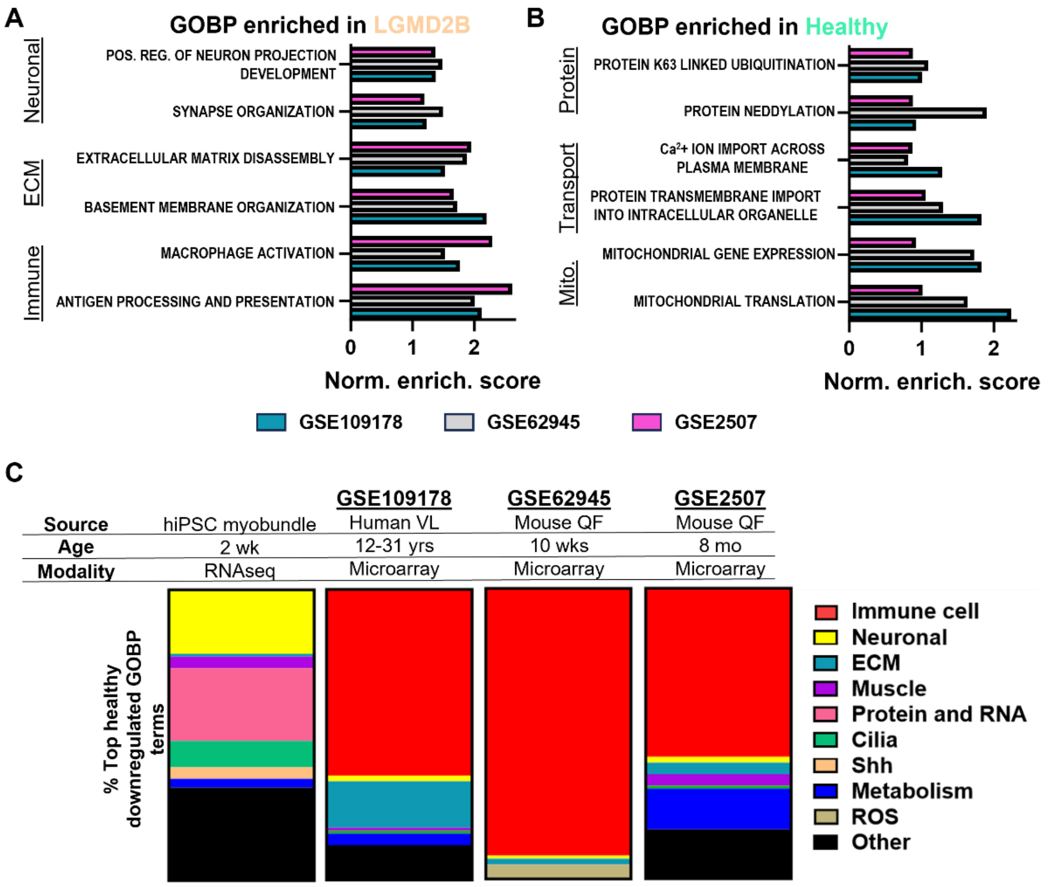

Figure S7. Gene ontology analysis of human and mouse myobundle and microarray data. (**A,B**) Representative gene ontology (GO) terms significantly enriched in (**A**) LGMD2B and (**B**) healthy human and mouse DNA microarray datasets. (**C**) Comparison of GOBP terms grouped by biological function upregulated in LGMD2B vs. healthy muscle in myobundle RNA-seq and native human and mouse microarray datasets.

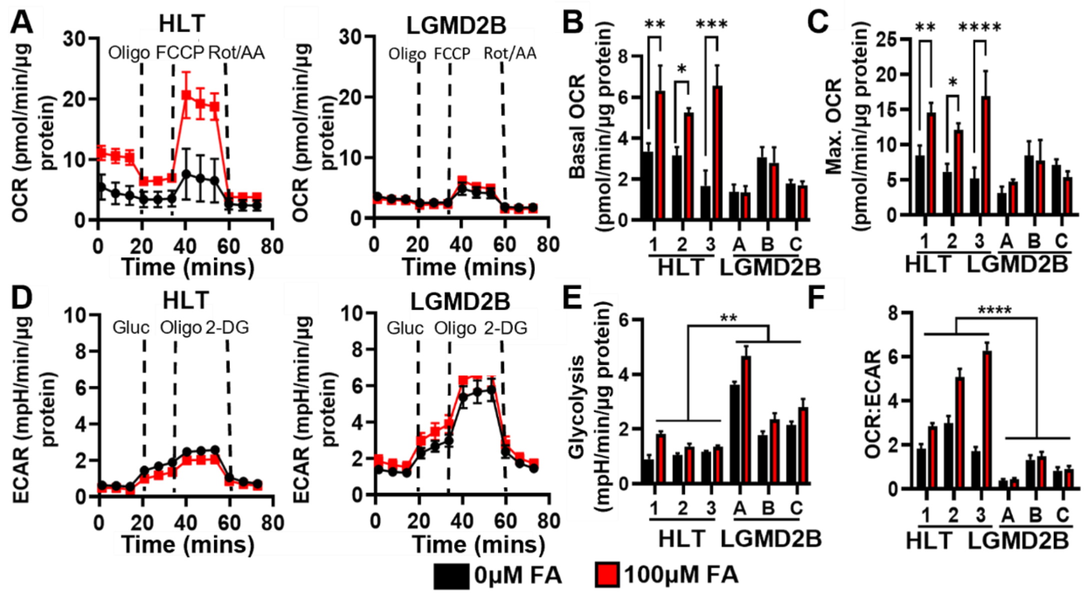

Figure S8. Cellular bioenergetics in healthy and LGMD2B myotubes. (**A**) Representative oxygen consumption rate (OCR) traces of healthy and LGMD2B myotubes cultured in 2D monolayers for 8 days and treated with 0 μM or 100 μM FA during the last 4 days of culture.

(**B,C**) Quantified (**C**) basal and (**D**) maximal OCR (N=1 differentiation, n=8-12 wells per group). (**D**) Representative extracellular acidification rate (ECAR) traces of healthy and LGMD2B myotubes cultured in 2D monolayers for 8 days and treated with 0 μM or 100 μM FA during the last 4 days of culture. (**E,F**) Quantified (**E**) basal ECAR and (**F**) OCR:ECAR ratio (N=1, n=8-12). *P<0.05, **p<0.01, ****P<0.001.

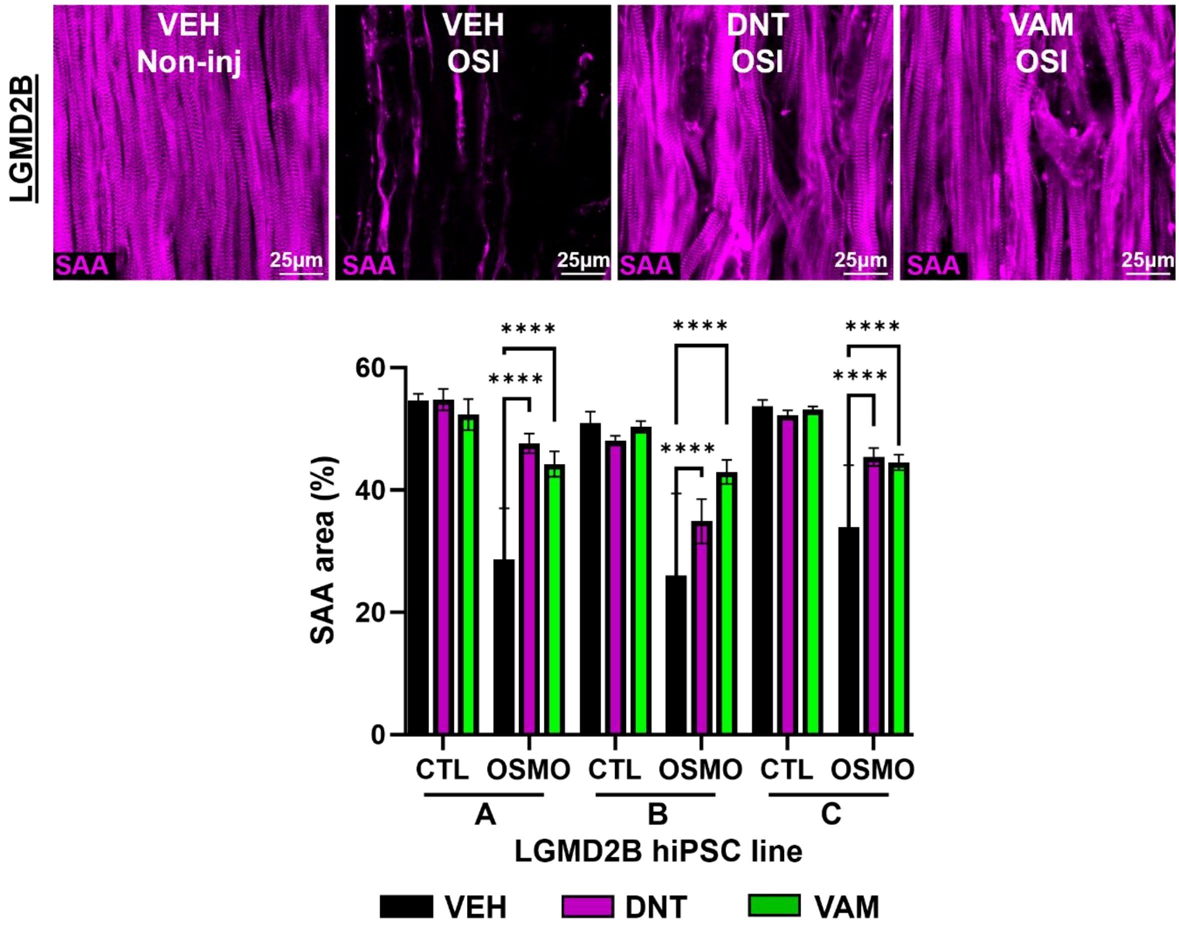

Figure S9. Membrane repair in dantrolene- and vamorolone-treated LGMD2B myobundles. LGMD2B myobundles were treated for 1 week with vehicle (VEH), dantrolene (DNT), or vamorolone (VAM) before undergoing osmotic shock injury (OSI). Representative whole-myobundle images of non-injured (non-inj) and OSI myobundles stained for sarcomeric alpha-actinin (SAA) with corresponding quantification of SAA^{+} area (N = 1 differentiation and n = 3 bundles per hiPSC line). ****P<0.001 vs. VEH.

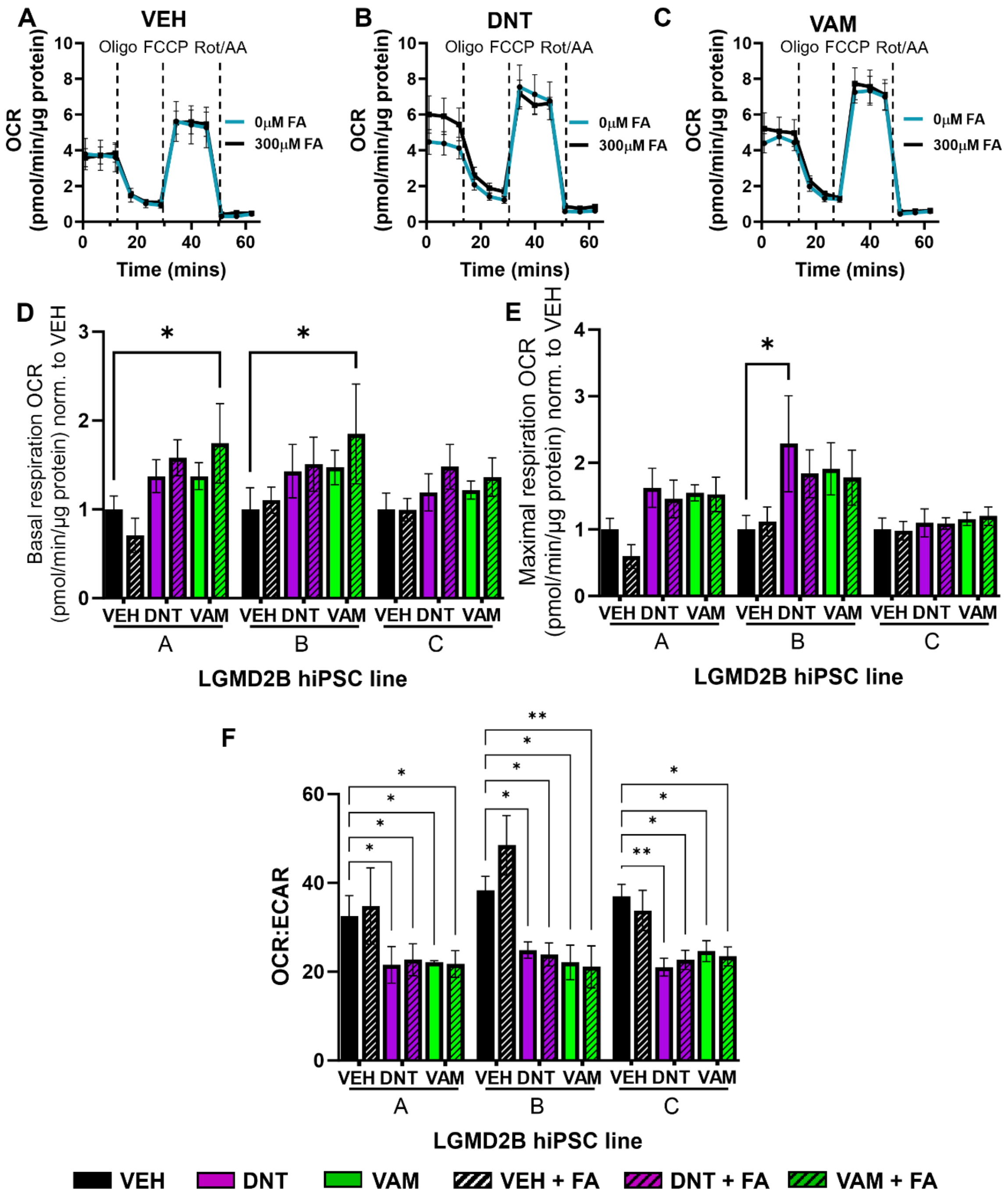

Figure S10. Cellular bioenergetics of dantrolene and vamorolone treated LGMD2B myobundles. (**A-C**) Representative seahorse oxygen consumption rate (OCR) traces in (**A**) vehicle (VEH), (**B**) dantrolene (DNT), and (**C**) vamorolone (VAM) treated LGMD2B myobundles with or without 300 μM fatty acids (FA). Corresponding quantifications of (**D**) basal respiration, (**E**) maximal respiration, and (**F**) OCR:ECAR (n = 5-10 myobundles per group). *P<0.05, **P<0.01 vs. VEH.

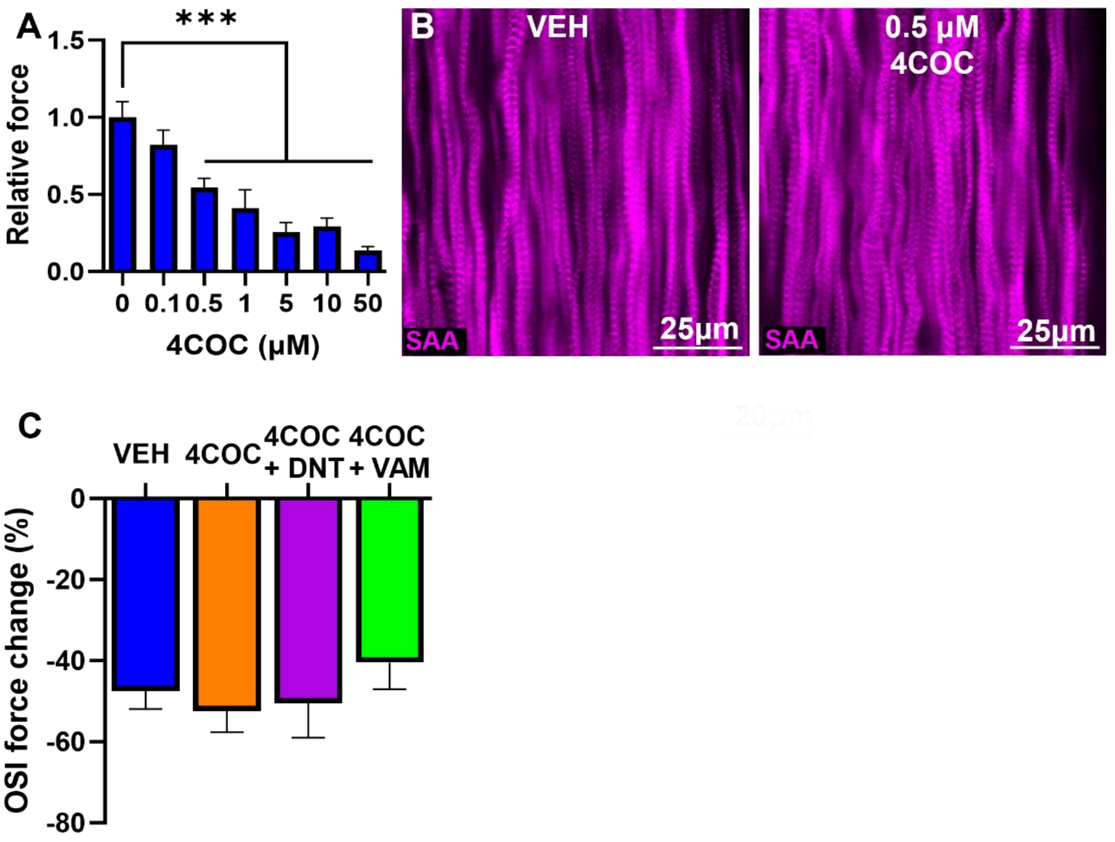

Figure S11. Effects of pharmacologically induced RyR leak on healthy myobundles. (**A**) Dose-dependent effect of 4COC on myobundle force generation (N = 1, n=3-4 per group). (**B**) Representative staining of sarcomeric alpha actinin (SAA) in vehicle (VEH) and 0.5 µM 4COC treated tissues. (**C**) Quantification of force change following OSI injury (N = 1 differentiation and n = 4-5 myobundles per group).

Media and solutions	Formulation
Neutralizing media	Low glucose DMEM supplemented with DNaseI (20 µg/mL, Sigma) and 50% FBS (Hyclone)
Sorting solution	E6 media supplemented with Y27632 (10 µM, Tocris), Doxycycline (1 µg/mL, Sigma), DNaseI (20 µg/mL, Sigma), 1% penicillin/streptomycin (Thermo)
Collecting solution	FBS (Hyclone) supplemented with Y27632 (10 µM, Tocris), Doxycycline (1 µg/mL, Sigma), 1% penicillin/streptomycin (Thermo)
Expansion media	Low glucose DMEM (Thermo) supplemented with 10% FBS (Hyclone), hEGF (10 µg/mL), Dexamethasone (0.4 µg/mL) Doxycycline (1 µg/mL, Sigma), 1% penicillin/streptomycin (Thermo)
Differentiation media 1	Low glucose DMEM (Thermo) supplemented with 1x N2 supplement (Thermo), penicillin G (100 unit/mL, Sigma), 2 mg/ml aminocaproic acid (Sigma), 0.1 µM smoothened agonist (Cayman chemical), EGF (2 ng/ml, Peprotech), LY209314 (Cayman chemical)
Differentiation media 2	Low glucose DMEM (Thermo) supplemented with 1x N2 supplement (Thermo), penicillin G (100 unit/mL, Sigma), 2 mg/ml aminocaproic acid (Sigma),
Differentiation media 3	Low glucose DMEM (Thermo) supplemented with 1x N2

	supplement (Thermo), penicillin G (100 unit/mL, Sigma), 2 mg/ml aminocaproic acid (Sigma), 5 nM T3 (Sigma), 200 µM carnitine, and 10 ng/ml biotin.
2D seahorse media	Seahorse XF DMEM, 5 mM glucose, 4 mM glutamine, 1 mM pyruvate, 0.1% ITS-X, 200 uM carnitine, and 300 uM fatty acids (1:1:1 oleic, linoleic, and palmitic acid)
3D seahorse media	Phys AA MEM (Thermo), 5 mM glucose, 2 mM glutamine, 0.2 mM pyruvate, 0.1% ITS-X, 200 µM carnitine, and 300 µM fatty acids (1:1:1 oleic, linoleic, and palmitic acid)
Cell/hydrogel mixture	12.5 million cells/mL in expansion media, bovine fibrinogen (4 mg/mL, Sigma), growth factor reduced matrigel (20% v/v, Corning), thrombin (0.2 U, Sigma)

Table S1. Cell culture media and solutions

Antibody	Application	Dilution	Company	Product No.
Dysferlin	IF	1:200	Abcam	Ab124684
Dysferlin	WB	1:500	Leica	NCL-Hamlet-2
Fast myosin heavy chain	IF	1:200	DSHB	F59
Myosin heavy chain	WB	1:1000	DSHB	MF20
Sarcomeric alpha actinin	IF, WB	IF (1:200)<br>WB (1:1000)	Sigma	A7732
Myomesin 2	IF	1:200	DSHB	mMaC myomesin B4
Titin	IF	1:200	DSHB	9D10
Pax7	IF	1:200	DSHB	Pax7
Myf5	IF	1:200	SCBT	Sc-302
Myogenin	IF	1:200	Abcam	Ab124800
RyR1	WB	1:1000	Thermo	MA3-925
DHPRa	WB	1:500	DSHB	IIC12D4
F-actin 488	IF	1:400	Thermo	A12379
DAPI	IF	1:400	Sigma	D1306
LipidSpot 610	IF	1:500	Biotium	70069

Table S2. Antibody information

Movie S1. Representative 2 week healthy and LGMD2B myobundle calcium transients.
